# Nonlinear flexibility effects on flight dynamics of high-aspect-ratio wings

**DOI:** 10.1186/s44147-026-01183-4

**Published:** 2026-08-25

**Authors:** Nikolaos D. Tantaroudas, Ilias Karachalios

**Affiliations:** 1 Institute of Communications and Computer Systems (ICCS), 9 Iroon Politechniou Street, Zografou, Athens 15773 Greece; 2 Department of Environment, School of Technology, University of Thessaly, Larissa, Larissa Greece

**Keywords:** Aeroelasticity, Flight dynamics, Geometric nonlinearity, High-aspect-ratio wings, HALE aircraft, Flexible aircraft

## Abstract

This paper quantifies, in a systematic parametric fashion, the manner in which geometric nonlinearity and structural flexibility alter the flight dynamic characteristics of high-aspect-ratio wings representative of high-altitude long-endurance (HALE) aircraft. A coupled aeroelastic–flight dynamic model is assembled from established constituent formulations—a geometrically-exact beam representation of the structural dynamics, unsteady two-dimensional strip theory for the aerodynamic loading, and quaternion-based rigid-body equations for the flight mechanics—into a monolithic system in which consistent load and motion transfer is enforced at every integration time step. The contribution lies not in new modelling methodology but in the systematic single-parameter investigation this framework enables: the wing stiffness is varied continuously across four orders of magnitude through a non-dimensional flexibility parameter, spanning from effectively rigid to highly flexible configurations, with planform, mass distribution, and flight condition held fixed. The study reveals that increasing flexibility profoundly modifies the trim conditions, flutter boundaries, and the dynamic gust response. In particular, over the flexibility range in which the trimmed state is physically valid, the trim angle of attack is found to vary non-monotonically with flexibility: torsional wash-in initially lowers the required trim angle, whereas increasing static deformation produces an effective dihedral that reorients the local lift vectors and, as the wing bends further, demands higher trim angles to maintain vertical force equilibrium. The degradation of flutter speed with increasing flexibility is quantified through an aeroelastic mode-tracking analysis and is shown to depend on whether the pre-stressed equilibrium state is incorporated, with the pre-stress correction reaching about three per cent at the upper end of the physically valid flexibility range. Consistent with previously published findings, flexibility is found to erode longitudinal flight-dynamic (phugoid) stability so that active control becomes essential; the present framework, constructed and validated for the aeroelastic response, treats this trend qualitatively and does not claim quantitative flight-dynamic eigenvalues, which require a dedicated flight-mechanics model. The framework is validated against established HALE aircraft benchmark cases, demonstrating close agreement in structural natural frequencies, flutter and divergence speeds, and rigid-aircraft trim; the static aeroelastic deflections are conservative (overpredicted) with respect to three-dimensional aerodynamic references, a documented consequence of the two-dimensional strip aerodynamics that is discussed openly in the paper. The results provide quantitative criteria for identifying the conditions under which linear analysis remains adequate and the circumstances under which fully coupled nonlinear computational tools become indispensable.

## Introduction

The evolution of contemporary high-altitude long-endurance (HALE) and solar-powered unmanned aerial vehicle (UAV) platforms has pushed aircraft design well beyond the boundaries within which traditional aeroelastic and flight dynamic analysis methodologies remain valid. Vehicles such as NASA’s Helios prototype [1], the Airbus Zephyr series, and a growing number of conceptual solar-powered aircraft configurations employ high-aspect-ratio wings with remarkably low structural weight fractions, giving rise to wing-tip deflections that can exceed 25% of the semi-span under normal operational conditions [2, 3]. At such deformation levels, the conventional engineering practice of treating the structural, aerodynamic, and flight dynamic behaviours as separate, linearised problems becomes fundamentally inadequate. The pronounced interdependence among these three physical disciplines demands an integrated computational treatment capable of faithfully resolving the coupled, nonlinear physics that govern the aircraft response throughout its operating envelope. The need for such integrated analysis has become increasingly recognised following several high-profile incidents involving very flexible aircraft.

The catastrophic loss of the Helios prototype in June 2003 [1] served as a stark reminder of the critical need for integrated aeroelastic–flight dynamic analysis tools that can capture the nonlinear interplay between large structural deformations and the rigid-body dynamics of very flexible aircraft (VFA). The post-accident investigation traced the failure, in part, to an unanticipated interaction among structural flexibility, aerodynamic forces, and the flight control system, making it clear that these coupled phenomena cannot be reliably forecast using standard decoupled methods. The official mishap report emphasised that “the existing analytical tools used during development of the Helios configuration were not adequate to predict the vehicle’s stability characteristics under the atmospheric conditions encountered,” a conclusion that has provided strong impetus for the present and related research efforts. This event underscored that conservative design margins alone are insufficient when fundamental coupling mechanisms are neglected.

Substantial progress has been achieved in constructing computational frameworks tailored to VFA analysis. Patil, Hodges, and Cesnik [2, 4] were among the first to employ geometrically-exact beam formulations in conjunction with finite-state aerodynamics for HALE aircraft, establishing that flexibility exerts a profound influence on flight dynamic stability, particularly on the phugoid mode. In a landmark contribution, Patil and Hodges [5] demonstrated that the phugoid mode of a representative HALE configuration becomes unstable once wing flexibility is taken into account—a result that conventional rigid-body flight dynamic analysis entirely fails to predict. These early studies set the stage for a sustained research effort in the field. Hodges [6, 7] formulated the variational-asymptotic beam sectional analysis (VABS) together with the geometrically-exact intrinsic beam theory that has since formed the theoretical backbone of much of the subsequent work. Cesnik and Brown [8] broadened these concepts to encompass active aeroelastic tailoring of HALE wings, while Shearer and Cesnik [9] constructed a comprehensive nonlinear flight dynamics simulation capability specifically designed for very flexible aircraft. In a closely related line of work, Arena et al. [10] developed a fully nonlinear aeroelastic formulation for flexible high-aspect-ratio wings that combines geometrically exact wing kinematics with an unsteady aerodynamic model incorporating dynamic stall, and employed it to compute pre-stressed aeroelastic equilibria and the ensuing post-flutter limit-cycle behaviour; their results demonstrated that both the flutter onset and the post-critical response are strongly conditioned by the nonlinear equilibrium state about which the dynamics evolve, a conclusion that directly informs the pre-stressed flutter analysis undertaken in the present study.

The sophistication of aerodynamic modelling has likewise advanced considerably over the past two decades. Murua et al. [11, 12] interfaced an unsteady vortex-lattice method (UVLM) with a geometrically-nonlinear beam model, thereby delivering improved predictions of unsteady wake effects on the stability of flexible aircraft. Their research revealed that three-dimensional aerodynamic phenomena can modify flutter predictions by 5–10% relative to strip-theory-based approaches, with the magnitude of the discrepancy depending on the aspect ratio and the reduced frequency range under consideration. Such differences can be consequential for design-level flutter margin assessment. Hesse and Palacios [13, 14] devised reduced-order models for the geometrically consistent coupling of nonlinear beams with unsteady aerodynamics, and showed that preserving geometric consistency between the structural and aerodynamic reference frames is of paramount importance. Their work demonstrated that inconsistent linearisation—whereby the structure is linearised about the deformed state while the aerodynamic geometry remains based on the undeformed configuration—can introduce errors whose magnitude is comparable to those arising from neglecting nonlinearity altogether.

Tantaroudas and Da Ronch [3, 15] constructed nonlinear reduced-order aeroservoelastic models for very flexible aircraft, enabling computationally efficient control design for systems possessing thousands of degrees of freedom, and subsequently extended the methodology to control design for flexible free-flying aircraft [16]. Da Ronch et al. [17] undertook a systematic assessment of the influence of aerodynamic modelling fidelity on the prediction of manoeuvring aircraft response, comparing strip theory, doublet-lattice, UVLM, and RANS-based approaches across a spectrum of flexible configurations. The complete coupled aeroelastic–flight dynamic framework upon which the present work is built is documented in the companion paper [18].

More recent investigations have pursued coupling with higher-fidelity aerodynamic solvers and advanced reduced-order modelling. Wang et al. [19] studied CFD/CSD coupling for high-aspect-ratio flexible wings, highlighting the significance of viscous effects at elevated angles of attack while simultaneously confirming that inviscid methodologies deliver acceptable accuracy at moderate incidence angles. This finding has guided the selection of aerodynamic methods in subsequent parametric studies. Deskos et al. [20] assembled a thorough review of computational methods for flexible aircraft aeroelasticity, delineating the trade-offs among modelling fidelity, computational expense, and applicability to parametric design investigations. Su and Cesnik [21] examined the nonlinear aeroelasticity of blended-wing-body configurations, thereby extending the well-established beam-based methodologies to unconventional planform geometries. Artola et al. [22] demonstrated the viability of aeroelastic control and state estimation using a minimal nonlinear modal description combined with moving-horizon estimation and model-predictive control strategies. Goizueta et al. [23] introduced adaptive sampling techniques for interpolation of parametric reduced-order aeroelastic models across multiple flight conditions and design points. Riso and Cesnik [24] conducted a systematic assessment of the accuracy of low-order modelling approaches for aeroelastic predictions of very flexible wings, confirming that beam-based ROM frameworks are well suited to the large-deformation regime. In a complementary study, the same authors isolated the individual geometrically nonlinear mechanisms—nonlinear structural kinematics and follower aerodynamic loading—that govern wing aeroelastic dynamics at large static deflections, using the Pazy wing benchmark to quantify when each mechanism must be retained in the analysis [25]; their findings on the role of the nonlinear equilibrium state in conditioning the linearised dynamics are directly relevant to the flexibility study performed here. Del Carré and Palacios [26] developed efficient time-domain simulation techniques for nonlinear aeroelastic problems, and this line of development has been consolidated into SHARPy [27], an established open-source nonlinear aeroelastic simulation environment for very flexible aircraft and wind turbines that couples geometrically-exact composite beam dynamics with an unsteady vortex-lattice aerodynamic model, and which provides a valuable community reference implementation against which frameworks of the kind employed here can be cross-checked. A comprehensive treatment of the coupled flight mechanics, aeroelasticity, and control of flexible aircraft has recently been provided in the monograph by Palacios and Cesnik [28].

The formulation of active control strategies for very flexible aircraft has progressed in tandem with advances in modelling capability. Tantaroudas et al. [29] developed an adaptive aeroelastic control approach built upon nonlinear reduced-order models for gust load alleviation, and demonstrated that the method is robust to variations in both flight condition and structural parameters. Da Ronch et al. [30] designed a nonlinear controller for flutter suppression and validated it through wind tunnel testing, thereby bridging the divide between numerical prediction and experimental demonstration; a comprehensive survey of aircraft active flutter suppression technology and its maturation needs is provided by Livne [31]. Most recently, Gao et al. [32] developed a gust load alleviation framework for flexible flying wings using linear parameter-varying modelling with model predictive control, and Wang et al. [33] presented an $\mathcal {H}_\infty$ control law for gust alleviation of a high-aspect-ratio flexible wing with experimental wind tunnel validation.

Notwithstanding these advances, a systematic parametric exploration of how the degree of structural flexibility influences the complete spectrum of flight dynamic behaviour—encompassing trim, stability, and dynamic gust response—remains scarce in the open literature. It should be stated clearly at the outset what the present work does and does not claim as novel. The individual physical mechanisms at play are well documented in the literature: the lift-vector rotation induced by large bending deformations and its effect on trim were identified by Patil et al. [2], the destabilisation of the phugoid mode by wing flexibility was established by Patil and Hodges [5], and the necessity of geometrically nonlinear, fully coupled analysis at large deflections has been demonstrated repeatedly [9, 10, 24, 25]. Likewise, the constituent models assembled here—geometrically-exact beam dynamics, finite-state unsteady strip theory, and quaternion-based rigid-body equations—are established formulations rather than new methodological developments. The contribution of this paper is instead a systematic quantification. Whereas the majority of published investigations concentrate on a single configuration at its nominal stiffness, or on a small number of discrete stiffness values, the present study varies a single non-dimensional stiffness parameter *σ * continuously across four orders of magnitude—holding the planform, mass distribution, and flight condition fixed—so that the trim condition, the aeroelastic stability (flutter and divergence, with the flutter boundary evaluated about both the undeformed and the pre-stressed equilibria), and the discrete-gust response can be compared like-for-like over the entire flexibility range within one consistent modelling framework; the longitudinal flight-dynamic (phugoid) trend is addressed qualitatively, in the light of the simplified free-flight representation discussed in Limitations section. The outcome is a set of quantitative regime boundaries, expressed in terms of *σ * and the associated tip-deflection ratios, that indicate when linear analysis suffices, when a nonlinear static (trim) analysis is required, and when fully coupled nonlinear simulation becomes indispensable. The study pursues four specific objectives: first, to validate the coupled framework against established HALE benchmarks, including natural frequencies, static deflections, and flutter speeds; second, to quantify how flexibility alters trim conditions, with particular attention to the lift-vector rotation mechanism; third, to determine the flutter speed degradation as a function of *σ *—contrasting linearisation about the undeformed and pre-stressed equilibrium states—and to identify the physical mechanisms responsible; and fourth, to characterise the dynamic gust response across the full range of flexibility and to distil the results into practical guidance on the selection of analysis fidelity for design.

The remainder of this paper is structured as follows. Coupled aeroelastic–flight dynamic model section describes the coupled aeroelastic–flight dynamic model, encompassing the structural, aerodynamic, and rigid-body subsystems together with their monolithic coupling, the nonlinear trim procedure, the linearisation and mode-tracking methodology, and the numerical implementation. Flexibility parameter study section defines the flexibility parameter study and the test aircraft configuration. Results section presents the convergence studies, the validation, and the parametric results. Discussion section examines the practical implications of the findings, Limitations section delineates the limitations of the study, and Conclusions section summarises the principal conclusions.

## Coupled aeroelastic–flight dynamic model

**Fig. 1:**
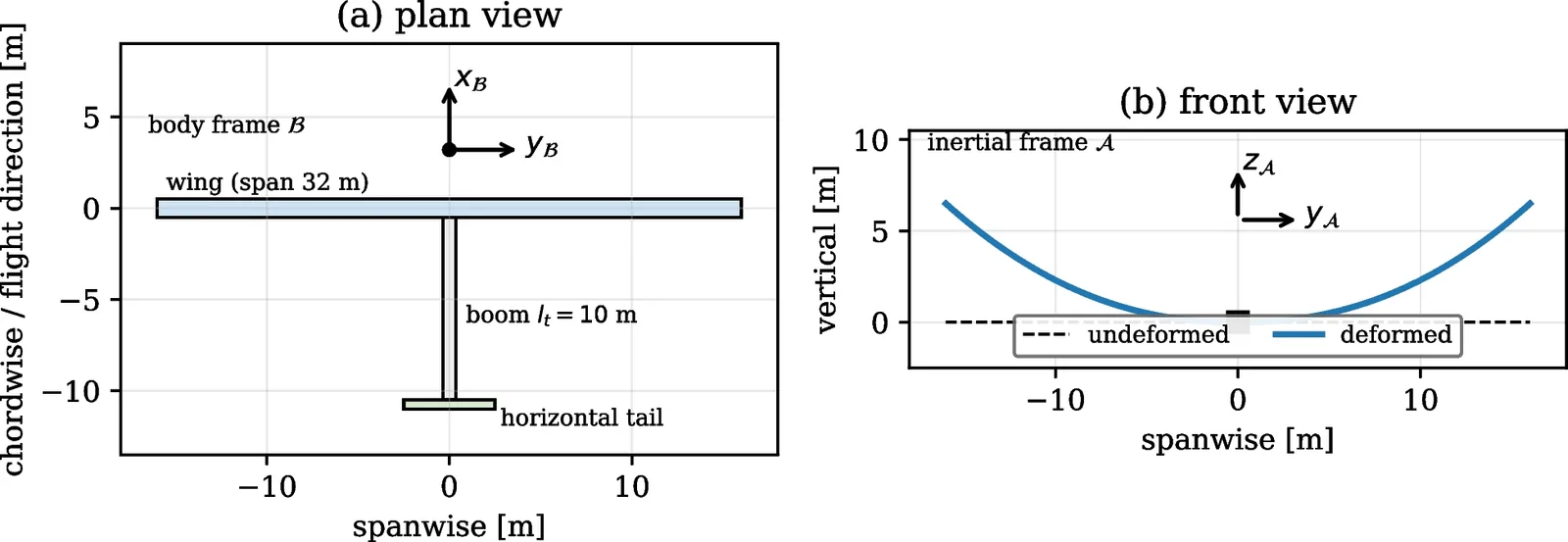
HALE aircraft geometry and coordinate-frame definitions: **a** plan view showing the body-fixed frame $\mathcal {B}$ (forward axis $x_{\mathcal {B}}$, spanwise axis $y_{\mathcal {B}}$), the wing, boom, and horizontal tail; **b** front view showing the inertial frame $\mathcal {A}$ and the deformed wing shape. The wing undergoes large bending deformation described by the geometrically nonlinear beam formulation

The coupled computational framework is composed of three subsystems: a geometrically-exact beam model for the structural response (Structural model: geometrically-exact beam theory section), an unsteady strip-theory aerodynamic model (Aerodynamic model: unsteady strip theory section), and a quaternion-based six-degree-of-freedom (6-DOF) flight dynamic model (Flight dynamic model: quaternion-based 6-DOF equations section). These components are monolithically coupled via consistent transformations of loads and kinematics (Monolithic coupling section), ensuring that all inter-disciplinary interactions are resolved within a single solution procedure. The nonlinear trim procedure, the linearisation about the trimmed state together with the mode-tracking methodology, and the numerical implementation are documented in Nonlinear trim procedure–Numerical implementation sections. The overall architecture is depicted schematically in Fig. 1, which illustrates the HALE aircraft geometry and the coordinate frames employed throughout the formulation.

### Structural model: geometrically-exact beam theory

The wing structure is represented as a one-dimensional beam capable of undergoing arbitrarily large displacements and rotations, following the geometrically-exact formulation [6, 34]. This methodology preserves the full geometric nonlinearity without introducing approximations, rendering it well-suited for the large deformations that characterise HALE aircraft wings. By treating the wing as a beam rather than a full three-dimensional solid, the computational cost is kept manageable while retaining the essential physics. Each cross-section of the beam is described by six degrees of freedom: three translations $(u_1, u_2, u_3)$ and three rotations $(\theta _1, \theta _2, \theta _3)$ defined relative to a body-fixed reference frame $\mathcal {B}$.

The position vector of a material point on the deformed beam reference line is given by

1 $$\begin{aligned} \boldsymbol{r}(s,t) = \boldsymbol{r}_0(s) + \boldsymbol{u}(s,t), \end{aligned}$$

where $\boldsymbol{r}_0(s)$ denotes the undeformed position, $\boldsymbol{u}(s,t)$ is the displacement vector, and *s* represents the curvilinear coordinate measured along the beam span. Although the deformed beam reference line is taken to be inextensible in the initial formulation, the extensional degree of freedom is retained within the stiffness matrix to ensure generality and accommodate cases where axial deformation may become relevant.

The orientation of each cross-section is characterised by a rotation tensor $\boldsymbol{C}^{Ba}(s,t)$ that maps vectors from the inertial frame $\mathcal {A}$ to the local beam frame $\mathcal {B}$. This tensor is parameterised by means of the Cartesian rotation vector $\boldsymbol{\psi }$, yielding

2 $$\begin{aligned} \boldsymbol{C}^{Ba} = \boldsymbol{I} + \frac{\sin \Vert \boldsymbol{\psi }\Vert }{\Vert \boldsymbol{\psi }\Vert }\widetilde{\boldsymbol{\psi }} + \frac{1 - \cos \Vert \boldsymbol{\psi }\Vert }{\Vert \boldsymbol{\psi }\Vert ^2}\widetilde{\boldsymbol{\psi }}^2, \end{aligned}$$

where $\widetilde{\boldsymbol{\psi }}$ signifies the skew-symmetric matrix associated with $\boldsymbol{\psi }$, and $\boldsymbol{I}$ is the *3× 3* identity matrix. This Rodrigues parameterisation circumvents the redundancy inherent in quaternion representations for local cross-sectional orientation while furnishing a singularity-free description for rotations up to *2π *, which comfortably exceeds the range of wing deformations encountered in the present study.

The strain measures—force strain $\boldsymbol{\gamma }$ and moment strain $\boldsymbol{\kappa }$—are defined according to

3 $$\begin{aligned} \boldsymbol{\gamma }(s,t)&= \boldsymbol{C}^{Ba}\left( \boldsymbol{r}_0' + \boldsymbol{u}'\right) - \boldsymbol{e}_1, \end{aligned}$$

4 $$\begin{aligned} \boldsymbol{\kappa }(s,t)&= \left( \boldsymbol{C}^{Ba}\right) '\boldsymbol{C}^{aB}\boldsymbol{e}_1 - \boldsymbol{\kappa }_0, \end{aligned}$$

where primes indicate derivatives with respect to *s*, $\boldsymbol{e}_1 = [1,0,0]^\top$ is the unit tangent vector of the undeformed beam, and $\boldsymbol{\kappa }_0$ represents the initial curvature. The force strain $\boldsymbol{\gamma }$ encapsulates axial extension and transverse shear deformation, while the moment strain $\boldsymbol{\kappa }$ captures bending about two perpendicular axes and torsion. Within the geometrically-exact framework, these strain measures are objective—that is, they remain invariant under superimposed rigid-body motions—a property that is essential for the consistent coupling with the flight dynamic equations.

The constitutive relation connects the generalised strains to the sectional forces $\boldsymbol{F}$ and moments $\boldsymbol{M}$ through the cross-sectional stiffness matrix:

5 $$\begin{aligned} \left\{ \begin{array}{c} \boldsymbol{F} \\ \boldsymbol{M} \end{array}\right\} = \left[ \begin{array}{cc} \boldsymbol{S}_{11} & \boldsymbol{S}_{12} \\ \boldsymbol{S}_{12}^\top & \boldsymbol{S}_{22} \end{array}\right] \left\{ \begin{array}{c} \boldsymbol{\gamma } \\ \boldsymbol{\kappa } \end{array}\right\} , \end{aligned}$$

where the constituent sub-matrices assume the explicit forms

6 $$\begin{aligned} \boldsymbol{S}_{11} = \left[ \begin{array}{ccc} EA & 0 & 0 \\ 0 & GA_2 & 0 \\ 0 & 0 & GA_3 \end{array}\right] , \quad \boldsymbol{S}_{22} = \left[ \begin{array}{ccc} GJ & 0 & 0 \\ 0 & EI_2 & 0 \\ 0 & 0 & EI_3 \end{array}\right] , \quad \boldsymbol{S}_{12} = \boldsymbol{0}, \end{aligned}$$

for an isotropic, symmetric cross-section devoid of structural coupling. In these expressions, *EA* represents the extensional stiffness, $GA_2$ and $GA_3$ are the shear stiffnesses, *GJ* is the torsional stiffness, and $EI_2$ and $EI_3$ are the bending stiffnesses about the two principal axes. For the HALE wing configuration examined in this work, $\boldsymbol{S}_{12} = \boldsymbol{0}$ (indicating no bend-twist coupling), although the formulation is general enough to accommodate non-zero coupling terms that would arise in composite or swept wing designs.

The governing equations of motion are derived from Hamilton’s principle. The virtual work of the internal and inertial forces produces the weak form:

7 $$\begin{aligned} \int _0^L \left[ \delta \boldsymbol{\gamma }^\top \boldsymbol{F} + \delta \boldsymbol{\kappa }^\top \boldsymbol{M}\right] \textrm{d}s + \int _0^L \left[ \delta \boldsymbol{u}^\top \mu \ddot{\boldsymbol{u}} + \delta \boldsymbol{\psi }^\top \boldsymbol{J}_\rho \ddot{\boldsymbol{\psi }}\right] \textrm{d}s = \int _0^L \delta \boldsymbol{u}^\top \boldsymbol{f}_{\textrm{ext}} \,\textrm{d}s, \end{aligned}$$

where *μ * denotes the mass per unit length, $\boldsymbol{J}_\rho$ is the cross-sectional rotational inertia matrix, and $\boldsymbol{f}_{\textrm{ext}}$ encompasses aerodynamic loads and gravitational forces. The beam is spatially discretised using two-noded finite elements with linear shape functions for both displacements and rotations. This discretisation yields the semi-discrete system

8 $$\begin{aligned} \boldsymbol{M}_s\,\ddot{\boldsymbol{\eta }} + \boldsymbol{C}_s(\boldsymbol{\eta },\dot{\boldsymbol{\eta }})\,\dot{\boldsymbol{\eta }} + \boldsymbol{K}_s(\boldsymbol{\eta })\,\boldsymbol{\eta } = \boldsymbol{f}_{\textrm{ext}}(\boldsymbol{\eta },\dot{\boldsymbol{\eta }},t), \end{aligned}$$

where $\boldsymbol{\eta }$ collects the nodal degrees of freedom, $\boldsymbol{M}_s$ is the consistent mass matrix, $\boldsymbol{C}_s$ is the velocity-dependent matrix collecting the gyroscopic and Coriolis contributions that arise from the rotating kinematic description, and $\boldsymbol{K}_s(\boldsymbol{\eta })$ is the tangent stiffness matrix. It is emphasised that $\boldsymbol{C}_s$ contains *no* material or structural damping: dissipation in the structure is deliberately neglected throughout this work ($\boldsymbol{C}_s$ vanishes identically when the rigid-body rates are zero, as in the clamped-wing analyses). No Rayleigh, modal, or hysteretic damping is applied at any point. This choice is conservative for the flutter and gust-response predictions—any physical structural dissipation would raise the flutter speed and accelerate the post-gust decay—and it ensures that all damping observed in the computed responses is of purely aerodynamic origin, which makes the modal damping trends reported in Results section directly interpretable as aerodynamic damping. The same assumption of zero inherent structural damping is adopted in the reference HALE benchmark studies [2, 5, 11], so the comparisons presented in HALE aircraft validation section are like-for-like in this respect. The explicit dependence of $\boldsymbol{K}_s$ on $\boldsymbol{\eta }$ embodies the geometric nonlinearity: the stiffness evolves with the current deformed configuration, a feature that is absent from linear structural models. The tangent stiffness matrix admits the decomposition

9 $$\begin{aligned} \boldsymbol{K}_s(\boldsymbol{\eta }) = \boldsymbol{K}_e + \boldsymbol{K}_g(\boldsymbol{\eta }), \end{aligned}$$

where $\boldsymbol{K}_e$ is the linear elastic stiffness and $\boldsymbol{K}_g(\boldsymbol{\eta })$ is the geometric stiffness (also referred to as the stress-stiffness or initial-stress stiffness), which captures the influence of the current internal force state on the overall structural stiffness. For wings that bend upward under aerodynamic loading, the geometric stiffness introduces a tension-stiffening phenomenon that elevates the effective bending stiffness in the deformed configuration. This effect is entirely absent from linear models and grows increasingly significant at large tip deflections [6, 34]. Its proper inclusion is therefore essential for accurate prediction of both static and dynamic behaviour of very flexible wings.

### Aerodynamic model: unsteady strip theory

The aerodynamic loads are evaluated using two-dimensional unsteady strip theory founded on the Theodorsen formulation [35, 36]. At each spanwise strip, the lift, pitching moment, and drag are calculated from the local effective angle of attack, which incorporates contributions from both the rigid-body motion and the elastic deformation of the wing. Although the strip theory approach is less accurate than fully three-dimensional methods for planforms with low aspect ratios, it has been shown to deliver adequate fidelity for HALE-type configurations whose aspect ratios exceed 20 [2, 17]. Its computational efficiency also makes it particularly attractive for the extensive parametric sweeps undertaken in this study.

The total lift per unit span is composed of circulatory and non-circulatory (apparent-mass) contributions:

10 $$\begin{aligned} L(s,t) = L_{\textrm{nc}}(s,t) + L_c(s,t). \end{aligned}$$

The non-circulatory lift per unit span represents the inertia of the fluid displaced by the oscillating aerofoil:

11 $$\begin{aligned} L_{\textrm{nc}}(s,t) = \pi \rho b^2 \left[ \ddot{h} + U\dot{\alpha } - b\,a\,\ddot{\alpha }\right] , \end{aligned}$$

where *ρ * is the air density, *b* is the semi-chord, *U* is the local freestream velocity, *α * is the effective angle of attack, *h* is the plunge displacement, and *a* is the elastic axis location (expressed in semi-chords measured from mid-chord). This term captures the added-mass effect that becomes important at high reduced frequencies and during rapid manoeuvres.

The circulatory lift per unit span takes the form

12 $$\begin{aligned} L_c(s,t) = 2\pi \rho U b\, C(k)\left[ \dot{h} + U\alpha + b\left( \tfrac{1}{2}-a\right) \dot{\alpha }\right] , \end{aligned}$$

where *C*(*k*) is the Theodorsen function evaluated at the reduced frequency *k = ω b/U*. The aerodynamic moment per unit span about the elastic axis is given by

13 $$\begin{aligned} M_{\textrm{ea}}(s,t)&= \pi \rho b^2\!\left[ -b\,a\,\ddot{h} - U b\!\left( \tfrac{1}{2}-a\right) \dot{\alpha } - b^2\!\left( \tfrac{1}{8}+a^2\right) \ddot{\alpha }\right] \nonumber \\&\quad + 2\pi \rho U b^2\!\left( a+\tfrac{1}{2}\right) C(k)\!\left[ \dot{h} + U\alpha + b\!\left( \tfrac{1}{2}-a\right) \dot{\alpha }\right] . \end{aligned}$$

For time-domain simulations, the frequency-domain Theodorsen function is replaced by its time-domain counterpart through the Wagner function $\Phi (\tau )$ [37, 38]:

14 $$\begin{aligned} \Phi (\tau ) = 1 - \psi _1 e^{-\varepsilon _1 \tau } - \psi _2 e^{-\varepsilon _2 \tau }, \end{aligned}$$

where *τ = Ut/b* is the non-dimensional time, and $\psi _1 = 0.165$, $\psi _2 = 0.335$, $\varepsilon _1 = 0.0455$, $\varepsilon _2 = 0.3$ are the R.T. Jones approximation coefficients [38]. This exponential approximation enables efficient computation while retaining high accuracy for the aerodynamic indicial response. The circulatory lift in the time domain is then expressed as a Duhamel convolution integral:

15 $$\begin{aligned} L_c(s,t) = 2\pi \rho U b \int _0^t \Phi (t-\tau )\,\frac{\textrm{d}w_{3/4}}{\textrm{d}\tau }\,\textrm{d}\tau , \end{aligned}$$

where $w_{3/4}$ denotes the downwash at the three-quarter chord point. The Duhamel integral is computed using an augmented-state approach that introduces two additional aerodynamic state variables per strip to represent the lag dynamics:

16 $$\begin{aligned} \dot{x}_1(s,t)&= -\frac{\varepsilon _1 U}{b} x_1(s,t) + w_{3/4}(s,t), \end{aligned}$$

17 $$\begin{aligned} \dot{x}_2(s,t)&= -\frac{\varepsilon _2 U}{b} x_2(s,t) + w_{3/4}(s,t), \end{aligned}$$

so that the circulatory lift can be expressed in closed form as

18 $$\begin{aligned} L_c(s,t) = 2\pi \rho U b\left[ (1-\psi _1-\psi _2)w_{3/4} + \frac{\varepsilon _1\psi _1 U}{b} x_1 + \frac{\varepsilon _2\psi _2 U}{b} x_2\right] . \end{aligned}$$

This augmented-state formulation eliminates the requirement to store and evaluate the entire convolution history, making the method computationally tractable for extended time-domain simulations involving many time steps.

For gust analysis, the Küssner function $\Psi (\tau )$ furnishes the indicial lift response to a sharp-edged gust [39, 40]:

19 $$\begin{aligned} \Psi (\tau ) = 1 - \phi _1 e^{-\beta _1 \tau } - \phi _2 e^{-\beta _2 \tau }, \end{aligned}$$

with coefficients $\phi _1 = 0.5792$, $\phi _2 = 0.4208$, $\beta _1 = 0.1393$, $\beta _2 = 1.802$ [40]. The lift induced by the gust is evaluated through a Duhamel integral analogous to (15), with the downwash replaced by the gust velocity component acting normal to the local chord. Two additional augmented states per strip are introduced to capture the gust dynamics, bringing the total count of aerodynamic state variables to four per strip. This relatively compact state representation keeps the overall system dimension manageable even for finely discretised wings.

The coordinate transformation between the aerodynamic frame $\mathcal {A}$ (aligned with the local freestream direction) and the beam cross-sectional frame $\mathcal {B}$ is accomplished through the rotation matrix $\boldsymbol{R}_c$:

20 $$\begin{aligned} \boldsymbol{v}^{(\mathcal {B})} = \boldsymbol{R}_c \, \boldsymbol{v}^{(\mathcal {A})}, \end{aligned}$$

which accounts for the local sweep, dihedral, and twist angles that arise from both the undeformed geometry and the elastic deformation. The effective freestream velocity at each strip is obtained by transforming the body-frame velocity through the quaternion-derived rotation matrix $\boldsymbol{R}_\zeta$:

21 $$\begin{aligned} \boldsymbol{V}_{\textrm{eff}}(s) = \boldsymbol{R}_\zeta ^\top \boldsymbol{V}_\infty - \boldsymbol{v}_{\textrm{body}}(s) - \dot{\boldsymbol{u}}(s), \end{aligned}$$

where $\boldsymbol{V}_\infty$ is the inertial freestream velocity, $\boldsymbol{v}_{\textrm{body}}(s)$ is the velocity at station *s* arising from rigid-body motion, and $\dot{\boldsymbol{u}}(s)$ is the elastic velocity. The effective angle of attack at each strip is subsequently determined from

22 $$\begin{aligned} \alpha _{\textrm{eff}}(s) = \arctan \left( \frac{V_{\textrm{eff},3}(s)}{V_{\textrm{eff},1}(s)}\right) , \end{aligned}$$

where $V_{\textrm{eff},1}$ and $V_{\textrm{eff},3}$ represent the chordwise and normal components of the effective velocity, respectively. This expression inherently incorporates contributions from rigid-body pitch, structural twist, and the geometric effect of wing bending, which rotates the local chord plane relative to the freestream.

The aerodynamic drag is modelled using a parabolic drag polar:

23 $$\begin{aligned} D(s,t) = \frac{1}{2}\rho V_{\textrm{eff}}^2 c \left( C_{D_0} + \frac{C_L^2}{\pi e_0 AR}\right) , \end{aligned}$$

where $C_{D_0} = 0.01$ is the zero-lift drag coefficient and $e_0 = 0.95$ is the Oswald efficiency factor. For the high-aspect-ratio wings considered in this study, the induced drag contribution is comparatively small at the low lift coefficients characteristic of cruise flight, but grows in importance at the elevated angles of attack necessitated by trim of very flexible configurations.

### Flight dynamic model: quaternion-based 6-DOF equations

The rigid-body dynamics of the aircraft are governed by the Newton–Euler equations expressed in the body-fixed frame. The translational equations of motion are

24 $$\begin{aligned} m\left( \dot{\boldsymbol{V}}_B + \boldsymbol{\omega }_B \times \boldsymbol{V}_B\right) = \boldsymbol{F}_{\textrm{aero}} + \boldsymbol{F}_{\textrm{grav}} + \boldsymbol{F}_{\textrm{thrust}}, \end{aligned}$$

where *m* is the total aircraft mass, $\boldsymbol{V}_B = [u, v, w]^\top$ is the velocity expressed in the body frame, $\boldsymbol{\omega }_B = [p, q, r]^\top$ is the angular velocity vector, and the right-hand side collects the aerodynamic, gravitational, and thrust forces resolved in the body frame. For a flexible aircraft, the total aerodynamic force is obtained by integrating the distributed strip loads over the deformed wing:

25 $$\begin{aligned} \boldsymbol{F}_{\textrm{aero}} = \int _{-L}^{L} \boldsymbol{C}^{Ba}(s) \begin{array}{c} -D(s) \\ 0 \\ L(s) \end{array} \textrm{d}s, \end{aligned}$$

where the rotation tensor $\boldsymbol{C}^{Ba}(s)$ transforms the local aerodynamic forces from the strip frame to the body frame, thereby naturally capturing the influence of wing deformation on the direction in which forces act. This integral formulation ensures that the lift vector rotation effect—whereby upward bending redirects part of the lift force laterally—is automatically included.

The rotational equations of motion are

26 $$\begin{aligned} \boldsymbol{J}\,\dot{\boldsymbol{\omega }}_B + \boldsymbol{\omega }_B \times \boldsymbol{J}\,\boldsymbol{\omega }_B = \boldsymbol{M}_{\textrm{aero}} + \boldsymbol{M}_{\textrm{thrust}}, \end{aligned}$$

where $\boldsymbol{J}$ is the inertia tensor of the aircraft. For flexible aircraft, the inertia tensor is configuration-dependent and must be updated at each time step to reflect the current deformed shape:

27 $$\begin{aligned} \boldsymbol{J}(t) = \boldsymbol{J}_0 + \Delta \boldsymbol{J}(\boldsymbol{\eta }(t)), \end{aligned}$$

where $\boldsymbol{J}_0$ is the undeformed inertia and $\Delta \boldsymbol{J}$ is the correction attributable to elastic deformation. This correction is evaluated from the displaced mass distribution according to

28 $$\begin{aligned} \Delta \boldsymbol{J}(\boldsymbol{\eta })&= \int _{-L}^{L} \mu (s)\!\left[ \Vert \boldsymbol{u}(s)\Vert ^2 \boldsymbol{I} - \boldsymbol{u}(s)\boldsymbol{u}(s)^\top \right] \textrm{d}s \nonumber \\&\quad + 2\int _{-L}^{L} \mu (s)\!\left[ \boldsymbol{r}_0(s)^\top \boldsymbol{u}(s)\,\boldsymbol{I} - \textrm{sym}\!\left( \boldsymbol{r}_0(s)\boldsymbol{u}(s)^\top \right) \right] \textrm{d}s, \end{aligned}$$

where $\textrm{sym}(\cdot )$ denotes the symmetric part of a matrix. For the very flexible configurations examined in this study (*σ > 2*), the magnitude of the inertia correction $\Delta \boldsymbol{J}$ can be substantial relative to the undeformed inertia, making its inclusion indispensable for reliable flight dynamic predictions. Neglecting this correction would introduce systematic errors in the predicted angular accelerations and, consequently, in the stability characteristics.

The attitude of the aircraft is parameterised using unit quaternions $\boldsymbol{q} = [q_0, q_1, q_2, q_3]^\top$ subject to the constraint $\Vert \boldsymbol{q}\Vert = 1$, which circumvents the gimbal lock singularity that afflicts Euler angle representations [41]. The quaternion kinematic equation takes the form

29 $$\begin{aligned} \dot{\boldsymbol{q}} = \frac{1}{2}\boldsymbol{\Omega }\,\boldsymbol{q}, \quad \boldsymbol{\Omega } = \left[ \begin{array}{cccc} 0 & -p & -q & -r \\ p & 0 & r & -q \\ q & -r & 0 & p \\ r & q & -p & 0 \end{array}\right] . \end{aligned}$$

The rotation matrix that maps vectors from the inertial frame to the body frame is reconstructed from the quaternion components as

30 $$\begin{aligned} \boldsymbol{R}_\zeta = \left[ \begin{array}{ccc} 1-2(q_2^2+q_3^2) & 2(q_1 q_2 - q_0 q_3) & 2(q_1 q_3 + q_0 q_2) \\ 2(q_1 q_2 + q_0 q_3) & 1-2(q_1^2+q_3^2) & 2(q_2 q_3 - q_0 q_1) \\ 2(q_1 q_3 - q_0 q_2) & 2(q_2 q_3 + q_0 q_1) & 1-2(q_1^2+q_2^2) \end{array}\right] . \end{aligned}$$

The position of the centre of gravity in the inertial frame is propagated by the kinematic relation

31 $$\begin{aligned} \dot{\boldsymbol{p}}_{\textrm{cg}} = \boldsymbol{R}_\zeta ^\top \boldsymbol{V}_B, \end{aligned}$$

which furnishes the altitude and ground-track information required for evaluating the gravitational force and for assessing the trajectory response during gust encounters. Tracking the inertial position is also necessary for computing the gust velocity at the aircraft location when spatially varying gust profiles are considered.

### Monolithic coupling

The three subsystems are assembled into a single monolithic system to ensure that all coupling effects are treated simultaneously. Defining the composite state vector $\boldsymbol{x} = [\boldsymbol{\eta }^\top , \dot{\boldsymbol{\eta }}^\top , \boldsymbol{x}_a^\top , \boldsymbol{V}_B^\top , \boldsymbol{\omega }_B^\top , \boldsymbol{q}^\top , \boldsymbol{p}_{\textrm{cg}}^\top ]^\top$, where $\boldsymbol{x}_a$ collects the augmented aerodynamic states, the coupled equations of motion are cast in the form

32 $$\begin{aligned} \boldsymbol{M}_T(\boldsymbol{x})\,\dot{\boldsymbol{x}} + \boldsymbol{C}_T(\boldsymbol{x})\,\boldsymbol{x} + \boldsymbol{K}_T(\boldsymbol{x}) = \boldsymbol{f}_T(\boldsymbol{x},t), \end{aligned}$$

where $\boldsymbol{M}_T$, $\boldsymbol{C}_T$, and $\boldsymbol{K}_T$ are the coupled mass, damping, and stiffness matrices, respectively, and $\boldsymbol{f}_T$ is the total forcing vector. The coupling manifests through three distinct physical mechanisms. The first is aerodynamic coupling: the aerodynamic loads depend on both the elastic deformation—through the local angle of attack and the direction of the lift vector—and the rigid-body motion—through the freestream velocity and attitude. The second is inertial coupling: the elastic deformation alters the mass distribution and thereby modifies the inertia properties, influencing the rigid-body dynamics via the updated inertia tensor $\boldsymbol{J}(t)$. The third is kinematic coupling: the elastic velocities contribute to the total angular momentum of the aircraft, and conversely, the rigid-body rotation affects the effective boundary conditions imposed on the structural model. These three coupling pathways interact in a highly nonlinear fashion, particularly at high flexibility levels where the deformation is large.

The monolithic formulation handles all coupling terms implicitly within a single iteration loop, thereby circumventing the well-documented stability difficulties that plague staggered (partitioned) coupling schemes, particularly at low mass ratios where the added-mass effect is pronounced [13]. Implicit treatment is especially important for the present application because the wing mass per unit length is low relative to the aerodynamic forces.

The coupled system (32) is advanced in time using an implicit Newmark-*β * scheme (*β = 0.25*, *γ = 0.5*) combined with Newton–Raphson iteration at each time step to resolve the nonlinear structural terms. The tangent matrix employed in the Newton–Raphson iteration is

33 $$\begin{aligned} \boldsymbol{K}_T^{\textrm{tan}} = \frac{\partial \boldsymbol{R}}{\partial \boldsymbol{x}_{n+1}} = \frac{1}{\beta \Delta t^2}\boldsymbol{M}_T + \frac{\gamma }{\beta \Delta t}\boldsymbol{C}_T + \boldsymbol{K}_T + \frac{\partial \boldsymbol{f}_T}{\partial \boldsymbol{x}}, \end{aligned}$$

where $\boldsymbol{R}$ is the residual vector. Convergence is declared when the relative residual satisfies $\Vert \boldsymbol{R}\Vert /\Vert \boldsymbol{f}_T\Vert < 10^{-8}$. The time step is selected to adequately resolve the highest structural frequency of interest, typically *Δ t = 0.01* s [16], which provides sufficient temporal resolution of the pertinent structural modes.

### Nonlinear trim procedure

The reference state for all stability and gust-response analyses is the nonlinear trimmed equilibrium of the flexible aircraft in steady, straight and level flight. Because the wing deformation and the aerodynamic loads are mutually dependent, the trim problem couples the aircraft-level force and moment balance with the full nonlinear static aeroelastic equilibrium of the structure, and both must be converged simultaneously. The procedure employed here is standard in its individual ingredients; it is documented in full so that the results are reproducible.

The trim unknowns are collected in the vector

34 $$\begin{aligned} \boldsymbol{z} = \left[ \alpha _{\textrm{trim}},\; T_{\textrm{trim}},\; M_{\textrm{ctrl}}\right] ^\top , \end{aligned}$$

where $\alpha _{\textrm{trim}}$ is the pitch attitude of the body frame relative to the freestream, $T_{\textrm{trim}}$ is the thrust magnitude (assumed to act along the body *x*-axis through the centre of gravity), and $M_{\textrm{ctrl}}$ is an idealised trimming moment applied about the pitch axis at the centre of gravity. The latter represents the action of an idealised elevator on the horizontal tail (equivalently, a small centre-of-gravity shift), applied as a pure couple rather than through a modelled control-surface deflection; it is held constant in all subsequent stick-fixed stability and gust analyses. The trim residual enforces steady-state force and moment equilibrium in the longitudinal plane:

35 $$\begin{aligned} \boldsymbol{r}_{\textrm{trim}}(\boldsymbol{z}) = \left\{ \begin{array}{c} F_x^{\mathcal {A}}(\boldsymbol{z}, \bar{\boldsymbol{\eta }}(\boldsymbol{z})) \\ F_z^{\mathcal {A}}(\boldsymbol{z}, \bar{\boldsymbol{\eta }}(\boldsymbol{z})) - W \\ M_{y,\textrm{cg}}(\boldsymbol{z}, \bar{\boldsymbol{\eta }}(\boldsymbol{z})) \end{array}\right\} = \boldsymbol{0}, \end{aligned}$$

where $F_x^{\mathcal {A}}$ and $F_z^{\mathcal {A}}$ are the horizontal and vertical components of the total force (aerodynamic, gravitational, and propulsive) resolved in the inertial frame, *W* is the aircraft weight, and $M_{y,\textrm{cg}}$ is the total pitching moment about the centre of gravity. The notation $\bar{\boldsymbol{\eta }}(\boldsymbol{z})$ makes explicit that each evaluation of the trim residual requires an inner solution of the nonlinear static aeroelastic problem: for the current iterate $\boldsymbol{z}$, the structural equilibrium $\boldsymbol{K}_s(\bar{\boldsymbol{\eta }})\,\bar{\boldsymbol{\eta }} = \boldsymbol{f}_{\textrm{ext}}(\bar{\boldsymbol{\eta }}, \boldsymbol{z})$ is solved by a full Newton–Raphson iteration with the analytical tangent stiffness of Eq. (9), converged to a relative residual below $10^{-8}$. Load stepping (progressive application of the dynamic pressure in increments) is used to ensure robust convergence of the inner problem at high flexibility levels, where the equilibrium deflection is a large fraction of the semi-span.

The outer trim problem, Eq. (35), is solved by Newton’s method with the *3× 3* trim Jacobian $\partial \boldsymbol{r}_{\textrm{trim}}/\partial \boldsymbol{z}$ constructed column-wise by forward finite differences of the converged inner solutions; the outer iteration is declared converged when $\Vert \boldsymbol{r}_{\textrm{trim}}\Vert /W < 10^{-10}$. The rigid-aircraft trim solution is used as the initial guess at the lowest flexibility level, and a continuation strategy is employed across the *σ *-sweep: the converged trim state at each value of *σ * initialises the trim iteration at the next, which keeps the number of outer iterations small (typically three to five) even for the most flexible configurations. No convergence failures were encountered over the full parameter range investigated. It is emphasised that no element of this procedure is claimed as novel; the trim methodology follows established practice for very flexible aircraft [2, 9, 28], and its documentation here serves reproducibility.

### Linearisation about the trimmed equilibrium and mode tracking

For the linearised stability analysis, the nonlinear coupled system is linearised about the computed trimmed equilibrium $\bar{\boldsymbol{x}} = [\bar{\boldsymbol{\eta }}^\top , \boldsymbol{0}^\top , \bar{\boldsymbol{x}}_a^\top , \bar{\boldsymbol{V}}_B^\top , \boldsymbol{0}^\top , \bar{\boldsymbol{q}}^\top , \bar{\boldsymbol{p}}_{\textrm{cg}}^\top ]^\top$. Writing $\boldsymbol{x} = \bar{\boldsymbol{x}} + \Delta \boldsymbol{x}$ and retaining first-order terms yields the generalised eigenvalue problem

36 $$\begin{aligned} \left( \lambda ^2 \boldsymbol{M}_T + \lambda \boldsymbol{C}_T + \boldsymbol{K}_T^{\textrm{tan}}\right) \hat{\boldsymbol{x}} = \boldsymbol{0}, \end{aligned}$$

where $\lambda = \textrm{Re}(\lambda ) + i\omega$ are the complex eigenvalues and $\hat{\boldsymbol{x}}$ the corresponding eigenvectors; a negative real part $\textrm{Re}(\lambda )$ indicates a stable mode and the imaginary part *ω * provides the modal frequency. Four aspects of this linearisation require care, and each is made explicit below.

*(i) Tangent structural stiffness.* The structural contribution to $\boldsymbol{K}_T^{\textrm{tan}}$ is the tangent stiffness of Eq. (9) evaluated at the *deformed* equilibrium, $\boldsymbol{K}_s(\bar{\boldsymbol{\eta }}) = \boldsymbol{K}_e + \boldsymbol{K}_g(\bar{\boldsymbol{\eta }})$, so that the geometric (stress) stiffening associated with the pre-stressed state is retained. Linearising instead about the undeformed configuration replaces $\boldsymbol{K}_s(\bar{\boldsymbol{\eta }})$ by $\boldsymbol{K}_e$ and evaluates all aerodynamic coupling blocks on the undeformed geometry; both variants are computed in this work precisely to quantify the difference (Trim and flutter speed variation with flexibility section). Consistency between the structural and aerodynamic linearisation states is enforced throughout—the aerodynamic geometry (local dihedral, twist, and moment arms) is always evaluated on the same configuration as the structural tangent, avoiding the inconsistent-linearisation errors highlighted by Hesse and Palacios [14].

*(ii) Aerodynamic lag states.* The augmented Wagner states of Eqs. (16)–(17) are retained as independent states in the linearised system, with their equilibrium values $\bar{\boldsymbol{x}}_a$ obtained from the steady limit ($\dot{x}_i = 0$) at trim. Their governing equations are linear in the states but depend on the local geometry through the downwash $w_{3/4}$; the corresponding Jacobian blocks are therefore evaluated at the trimmed configuration. This finite-state treatment ensures that the linearised system remains a standard (non-transcendental) eigenvalue problem while retaining unsteady shed-wake memory, avoiding the frequency-domain matching inherent in classical *V*–*g* approaches.

*(iii) Quaternion states.* The unit-norm constraint $\Vert \boldsymbol{q}\Vert =1$ renders one quaternion degree of freedom redundant, and an unconstrained linearisation of Eq. (29) therefore produces one spurious zero eigenvalue associated with perturbations along the constraint direction, in addition to the physical zero eigenvalues associated with the invariance of the dynamics to horizontal position and heading. These structural zeros are identified analytically and excluded from the stability assessment. For the symmetric, longitudinal analyses reported in this paper the issue does not arise at all: the production stability computations employ a minimal parameterisation of the longitudinal dynamics in which the pitch attitude appears as a single state, so no constraint-induced spurious eigenvalue exists, and the physical eigenvalues are independent of whether the minimal or the quaternion parameterisation is used.

*(iv) Flight-dynamic derivatives.* The rigid-body sub-blocks of the linearised system are the flexible-aircraft counterparts of the classical stability derivatives: they are extracted numerically as directional derivatives of the total integrated loads, Eq. (25), with respect to the rigid-body states $(\boldsymbol{V}_B, \boldsymbol{\omega }_B, \boldsymbol{q})$, evaluated at the trimmed equilibrium with the elastic and aerodynamic lag states free to contribute. Because the loads are integrated over the deformed geometry, these derivatives automatically inherit the flexibility corrections (effective-dihedral lift rotation, aerodynamic-centre shift) whose consequences are examined in Results section. All Jacobian blocks other than the analytical structural tangent are computed by central finite differences of the nonlinear residual with componentwise step-size scaling; the adequacy of the differencing step was verified by confirming that the computed eigenvalues of the least stable modes are insensitive to a factor-of-ten variation of the step size in representative cases.

The eigenvalue problem, Eq. (36), recast in first-order state-space form, is solved with a dense QZ (generalised Schur) eigensolver, which is robust and returns the complete spectrum at the system sizes considered here; the stability assessment then concentrates on the eigenvalues closest to the imaginary axis, which correspond to the least stable modes of practical interest.

*Mode classification and tracking.* The eigensolutions of the coupled system do not separate a priori into “structural” and “flight-dynamic” families—at high flexibility the physical modes contain substantial contributions from both. Modes are classified by the kinetic-energy content of their eigenvectors, partitioned into rigid-body and elastic contributions: a mode is labelled a flight-dynamic mode (phugoid, short-period) when the rigid-body partition dominates, and an aeroelastic mode otherwise, with the dominant elastic constituent (first bending, first torsion, ...) identified by projection onto the in-vacuo mode shapes of the wing. As the flight speed or the flexibility parameter *σ * is swept, individual modes are tracked between adjacent parameter values by means of the modal assurance criterion (MAC) applied to the complex eigenvectors: each mode at step *k+1* is paired with the mode at step *k* that maximises the MAC, and a pairing is flagged for manual inspection when the maximum MAC falls below 0.7, which occurs only in the vicinity of modal coalescence. This procedure underpins the aeroelastic mode-tracking data reported in Flexibility effect on flight dynamic response section and permits an unambiguous statement of which aeroelastic mode becomes critical at each flexibility level.

### Numerical implementation

All computations reported in this paper were performed with an in-house simulation framework written in Python (NumPy/SciPy), developed by the authors; no third-party aeroelastic package was used. The framework implements the formulation of Structural model: geometrically-exact beam theory–Linearisation about the trimmed equilibrium and mode tracking sections directly: a finite-element module assembles the beam mass, damping, and tangent stiffness matrices (elastic and geometric contributions assembled analytically at the element level); an aerodynamic module evaluates the strip loads and the augmented-state dynamics; and a flight-dynamics module provides the rigid-body equations and quaternion kinematics. The three modules exchange loads and kinematics through the transformation matrices of Monolithic coupling section inside a single monolithic residual, so no partitioned sub-iteration is involved. For the symmetric, longitudinal analyses reported in this paper, the general formulation of Structural model: geometrically-exact beam theory section is specialised to the dominant deformation fields of the high-aspect-ratio wing: the nonlinear production model retains the out-of-plane (flatwise) bending field with exact large rotations, treated co-rotationally at the element level, together with the Saint-Venant torsion field and axial stretching, while chordwise (edgewise) bending—whose stiffness is two hundred times the flatwise value for this configuration (Table 1)—is retained only in the linear modal analyses. The follower character of the aerodynamic loading (lift and drag directions attached to the deformed section frames) is preserved exactly. The structural operators are assembled analytically at the element level; the monolithic stepping tangent is built from the analytical structural tangent together with finite-difference aerodynamic and rigid-body coupling blocks, factorised directly (LAPACK *LU*) and reused across Newton iterations and time steps until the convergence rate degrades, at which point it is refreshed. At the system sizes considered in this study (of order $10^2$–$10^3$ states) dense factorisation is both simpler and faster than sparse alternatives. Nonlinear static and trim solutions follow the procedures (and tolerances) of Nonlinear trim procedure section; time integration employs the Newmark-*β * scheme with Newton–Raphson sub-iteration as described in Monolithic coupling section, with a step accepted either when the residual satisfies the $10^{-8}$ relative tolerance or when the Newton update stalls at machine precision relative to the state norm (the latter occurs only for the stiffest, near-rigid configurations, where the residual floor is set by the rounding noise of the large stiffness terms); and stability eigenproblems are solved as described in Linearisation about the trimmed equilibrium and mode tracking section. The spatial and temporal discretisations used for every result in this paper are supported by the convergence study reported in Spatial and temporal convergence section, and the implementation is validated against independent published benchmarks in HALE aircraft validation section. The source code and the scripts that generate every figure and table in this paper are available from the corresponding author on reasonable request.

**Table 1:** Baseline HALE aircraft properties (after Patil et al. [2])

Property	Value	Unit
Semi-span, *L*	16	m
Total span	32	m
Chord, *c*	1	m
Aspect ratio, *AR*	32	–
Mass per unit length, *μ *	0.75	kg/m
Bending stiffness, $EI_2$	$2\times 10^4$	N m^2^
Bending stiffness, $EI_3$	$4\times 10^6$	N m^2^
Torsional stiffness, *GJ*	$1\times 10^4$	N m^2^
Moment of inertia (torsion), $j_t$	0.1	kg m
Elastic axis location	50% chord	–
Centre of gravity location	50% chord	–
Fuselage mass	50	kg
Payload	0	kg
Horizontal tail area, $S_t$	2.5	m^2^
Horizontal tail arm, $l_t$	10	m
Tail lift-curve slope	*2π *	rad^-1^

## Flexibility parameter study

### Definition of the non-dimensional stiffness parameter

In order to systematically explore the influence of structural flexibility on flight dynamics, a non-dimensional stiffness parameter *σ * is introduced and defined as

37 $$\begin{aligned} \sigma = \frac{EI_{\textrm{ref}}}{EI_{\textrm{actual}}}, \end{aligned}$$

where $EI_{\textrm{ref}}$ is a reference bending stiffness corresponding to a baseline HALE wing configuration, and $EI_{\textrm{actual}}$ is the actual bending stiffness employed in the analysis. A value of *σ = 0.001* corresponds to an essentially rigid wing (possessing actual stiffness 1000 times the reference), while *σ = 4* represents a highly flexible wing (with actual stiffness equal to one-quarter of the reference). This single parameter thus spans the full range from practically rigid to extremely compliant configurations. The parameter *σ * is applied uniformly to every entry of the stiffness matrix $\boldsymbol{S}$ in Eq. (5), so that the modified stiffness matrix becomes

38 $$\begin{aligned} \boldsymbol{S}(\sigma ) = \frac{1}{\sigma }\boldsymbol{S}_{\textrm{ref}}, \end{aligned}$$

thereby ensuring that the ratios among bending, torsional, and extensional stiffnesses remain invariant throughout the parametric study. This uniform scaling preserves the relative importance of different deformation modes and prevents artifacts that could arise from selectively softening individual stiffness components.

The physical significance of *σ * can be appreciated by relating it to the ratio of aerodynamic to elastic forces. For a uniform wing in steady flight, the tip deflection normalised by the semi-span scales approximately as

39 $$\begin{aligned} \frac{\delta _{\textrm{tip}}}{L} \approx \frac{q_\infty c C_L L^3}{8\,EI_2} = \frac{\sigma \, q_\infty c C_L L^3}{8\,EI_{2,\textrm{ref}}}, \end{aligned}$$

where $q_\infty = \frac{1}{2}\rho U^2$ is the dynamic pressure and $C_L$ is the wing lift coefficient. This scaling relationship reveals that *σ * is directly proportional to the static tip deflection for a given flight condition, establishing it as a natural and physically intuitive parameter for characterising the severity of flexibility effects.

It is important to note that the mass distribution is held constant across all values of *σ *. This deliberate choice isolates the influence of stiffness variation from that of mass changes, which would independently alter both the structural dynamics (through the natural frequencies) and the flight dynamics (through the aircraft weight and inertia). In a real aircraft design, reducing stiffness would ordinarily entail a concurrent reduction in structural mass, but the present parametric approach provides cleaner insight into the effect of stiffness alone, free from the confounding influence of mass variation.

### Baseline aircraft configuration

The baseline aircraft configuration adopted in this study is derived from the HALE model of Patil et al. [2], which has become a widely recognised standard benchmark for very flexible aircraft analysis. The aircraft comprises a straight, unswept wing with a rigid fuselage positioned at the mid-span and a rigid horizontal tail, modelled as a quasi-steady lifting surface at arm $l_t$ behind the wing elastic axis, which provides the pitch stiffness of the original benchmark; the tail is treated as massless so that the mass properties of Table 1 are unchanged. The geometric and structural properties are compiled in Table 1, and the overall layout is depicted in Fig. 1. The simplicity of this configuration, while idealised, allows for direct comparison with a large body of existing published results and isolates the fundamental physics of flexibility effects from configuration-specific complexities.

The flight conditions representative of HALE operations are: altitude $h = 20{,}000$ m, freestream velocity *U = 25* m/s, and air density *ρ = 0.0889* kg/m$^3$. The corresponding dynamic pressure is $q_\infty = 27.8$ Pa and the Reynolds number based on chord is $Re \approx 1.5 \times 10^5$.

The values of *σ * investigated in the parametric study are: $\{0.001, 0.01, 0.1, 0.5, 1.0, 1.5, 2.0, 2.5, 3.0, 3.5, 4.0\}$. For each value, three types of analyses are conducted: nonlinear static aeroelastic equilibrium (trim), linearised stability analysis about the trimmed state (encompassing flutter and flight dynamic modes), and nonlinear dynamic response to a discrete “1-minus-cosine” gust. Together, these analyses provide a comprehensive picture of how flexibility affects the full spectrum of aeroelastic and flight dynamic behaviour.

## Results

### Spatial and temporal convergence

Before any validation or parametric result is presented, the adequacy of the spatial discretisation and of the integration time step is established. Two convergence studies were performed on the baseline configuration of Table 1.

**Fig. 2:**
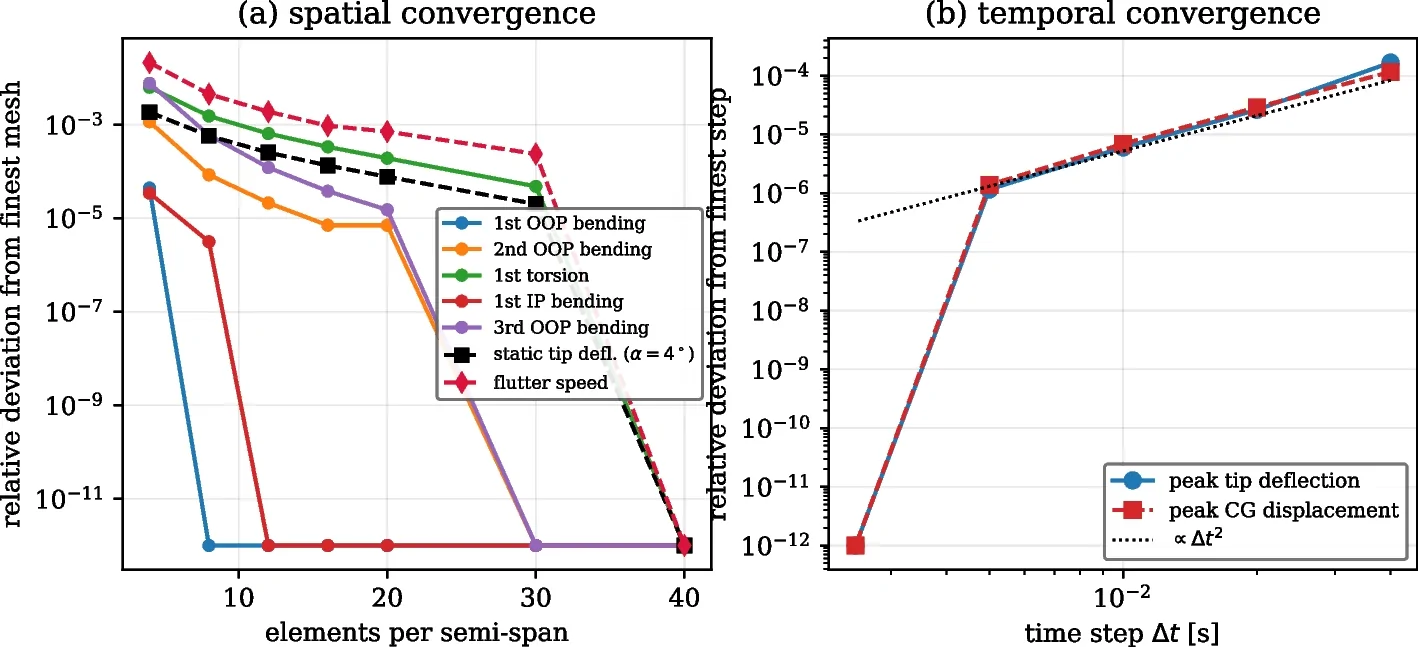
Convergence of the discretisations: **a** relative deviation from the finest mesh of the first five natural frequencies, the nonlinear static tip deflection at $\alpha =4^\circ$, and the flutter speed, versus the number of finite elements per semi-span; **b** relative deviation from the finest time step of the peak gust-response quantities at *σ =1*, with a $\Delta t^2$ reference slope

*Spatial convergence.* The number of finite elements per semi-span was varied from 4 to 40 and three classes of observable were monitored: the first five natural frequencies of the clamped wing, the nonlinear static tip deflection at *U = 25* m/s and $\alpha = 4^\circ$, and the flutter speed computed about the undeformed configuration. The results are summarised in Table 2 and Fig. 2a. All monitored quantities converge monotonically. At the production mesh of 20 elements, the five frequencies lie within 0.03% of their 40-element values (and within 0.03% of the exact beam-theory solutions, Table 4), the nonlinear static tip deflection is converged to within 0.02%, and the flutter speed to within 0.08% (32.68 m/s at $n_e = 20$ against 32.66 m/s at $n_e = 40$). The 20-element mesh is therefore adopted for all production analyses; even the coarse 12-element mesh used for the long time-domain simulations reproduces the flutter speed to 0.2% and the static tip deflection to 0.03%, which is ample for the trends studied here.

**Table 2:** Spatial convergence: first five natural frequencies (rad/s), nonlinear static tip deflection at $\alpha =4^\circ$, *U=25* m/s (m), and flutter speed about the undeformed state (m/s) versus the number of finite elements per semi-span

$\boldsymbol{n}_{\boldsymbol{e}}$	$\boldsymbol{\omega }_{\boldsymbol{1}}$	$\boldsymbol{\omega }_{\boldsymbol{2}}$	$\boldsymbol{\omega }_{\boldsymbol{3}}$	$\boldsymbol{\omega }_{\boldsymbol{4}}$	$\boldsymbol{\omega }_{\boldsymbol{5}}$	$\boldsymbol{\delta }_{\textbf{tip}}$	$\boldsymbol{V}_{\boldsymbol{f}}$
4	2.243	14.072	31.245	31.719	39.661	7.317	33.36
8	2.243	14.057	31.096	31.718	39.380	7.327	32.81
12	2.243	14.056	31.068	31.718	39.361	7.329	32.72
16	2.243	14.056	31.058	31.718	39.358	7.330	32.69
20	2.243	14.056	31.054	31.718	39.357	7.330	32.68
30	2.243	14.056	31.049	31.718	39.356	7.331	32.67
40	2.243	14.056	31.048	31.718	39.356	7.331	32.66

*Temporal convergence.* The gust-response simulation of Gust response: rigid vs. flexible section at *σ = 1* was repeated for a sequence of halved time steps from *Δ t = 0.04* s down to 0.0025 s, monitoring the peak wing-tip elastic deflection and the peak centre-of-gravity displacement over the initial response window. The results are given in Table 3 and Fig. 2b. Both monitored peaks are already reproduced to within *0.017%* at the coarsest step, and the production time step of *Δ t = 0.01* s is converged to five significant figures ($<10^{-3}\%$ change against the finest step); the average-acceleration Newmark scheme resolves the response with a large margin, as expected given that the highest frequency participating significantly in the response (*≈ 7* rad/s at this flexibility level) is sampled roughly ninety times per period at the production step.

**Table 3:** Temporal convergence of the *σ =1* gust-response simulation: peak wing-tip elastic deflection and peak centre-of-gravity displacement versus integration time step

$\boldsymbol{\Delta }\ \boldsymbol{t}$ [s]	peak $\boldsymbol{\delta }_{\textbf{tip}}$ [m]	peak $\boldsymbol{|\Delta z}_{\textbf{cg}}|$ [m]	change vs. finer [%]
0.04	4.4883	17.0926	0.014
0.02	4.4890	17.0911	0.002
0.01	4.4891	17.0907	*<0.001*
0.005	4.4891	17.0906	*<0.001*
0.0025	4.4891	17.0906	–

### HALE aircraft validation

Prior to the parametric study, the coupled framework is validated against previously published results for the HALE aircraft configuration specified in Table 1. Following the convergence study of Spatial and temporal convergence section, the wing is spatially discretised with 20 two-noded finite elements per semi-span (21 nodes; all six sectional degrees of freedom in the linear modal analyses, and the four degrees of freedom of the longitudinal specialisation—vertical and spanwise displacement, bending rotation, and twist—in the nonlinear analyses). For the symmetric longitudinal analyses reported here, the assembled first-order monolithic system comprises 249 states: the structural positions and velocities, two Wagner and two Küssner augmented aerodynamic states per strip (one strip per element), and the airspeed perturbation. This system size captures the relevant physics while remaining computationally tractable for the extensive parametric sweeps conducted subsequently.

#### Static aeroelastic validation

**Fig. 3:**
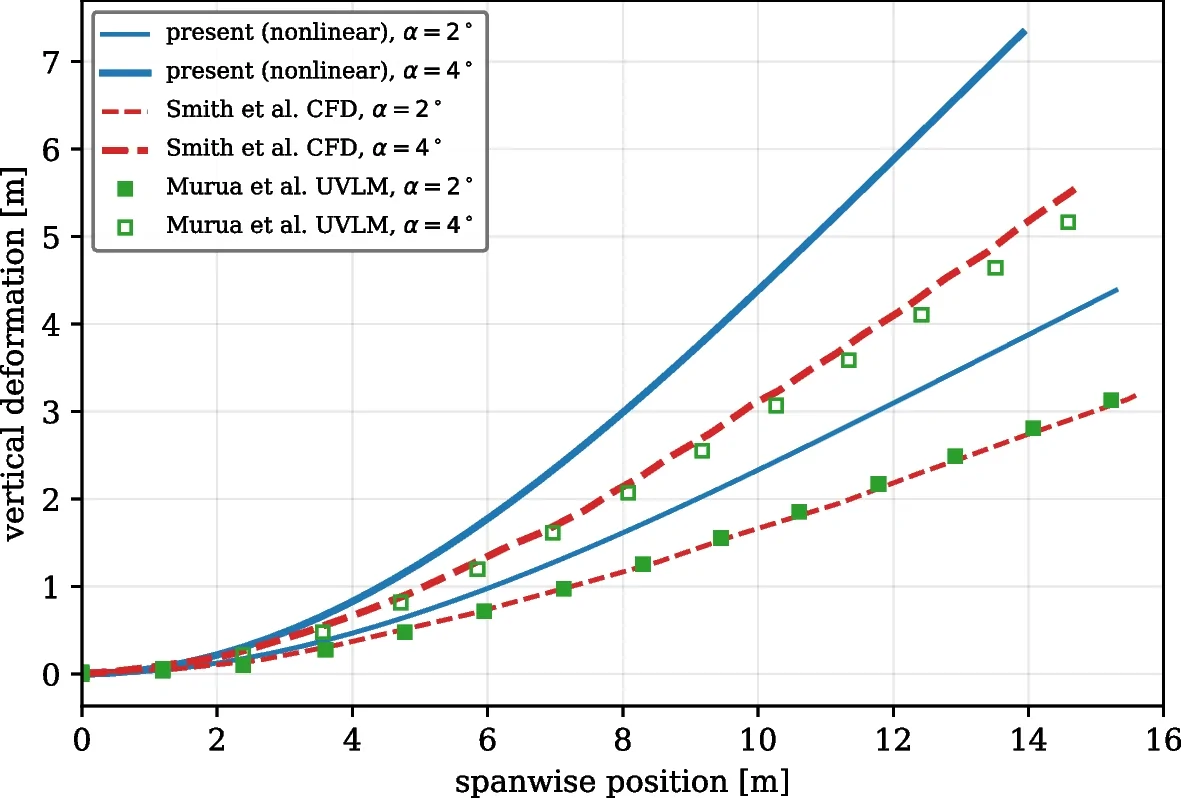
Static aeroelastic comparison: vertical wing deformation along the semi-span at $\alpha = 2^\circ$ and $\alpha = 4^\circ$ (*U = 25* m/s, no gravity), comparing the present strip-theory model with the CFD (Smith et al.) and UVLM data reported in Murua et al. [11]. The present two-dimensional aerodynamics overpredict the deflection (see text)

The nonlinear static aeroelastic solution is compared in Fig. 3 with the Euler-based aeroelastic computations of Smith et al. and the UVLM results of Murua et al., both as reported in [11], for the cantilevered wing at *U=25* m/s and root incidences of $2^\circ$ and $4^\circ$ (gravity excluded, as in the references). The spanwise distribution of the deformation and its strong growth between the two incidences are reproduced, but the tip deflection is overpredicted: the present model yields 4.39 m at $\alpha =2^\circ$ and 7.33 m at $\alpha =4^\circ$, against approximately 3.2 m and 5.6 m for the CFD and 3.2 m and 5.3 m for the UVLM. This conservatism has two identifiable sources, both rooted in the two-dimensional aerodynamic model. First, strip theory applies the full thin-aerofoil lift-curve slope along the entire span with no three-dimensional tip relief, so the outboard sections—whose loads dominate the root bending moment—are the most overloaded. Second, and dominant at this condition, the configuration operates at 45% of its torsional divergence dynamic pressure (Flutter speed section); the nose-up quarter-chord moment then amplifies the elastic wash-in strongly (computed tip twist $2.0^\circ$ and $3.4^\circ$ at the two incidences), and any overprediction of the sectional lift is compounded through this feedback loop before it reaches the bending response. Both mechanisms act in the same direction, so the present static aeroelastic loads are conservative with respect to three-dimensional aerodynamic references. The unsteady and integral quantities compared below (natural frequencies, flutter speed, divergence speed, and trim) show substantially closer agreement, and the parametric conclusions of this paper concern trends with the flexibility parameter rather than absolute deflection magnitudes; the conservatism is nonetheless stated plainly here and revisited in the Limitations section.

#### Natural frequencies and mode shapes

The natural frequencies obtained from the uncoupled structural model are compared with values reported in the literature in Table 4. The results computed by the present framework are in close agreement with those of Patil and Hodges [2], Murua et al. [11], and an exact beam theory solution, thereby confirming the fidelity of the structural discretisation.

**Table 4:** Natural frequencies (rad/s) of the HALE wing: comparison with published data

Mode	Present	Patil [2]	Exact beam theory
1st bending	2.24	2.24	2.24
2nd bending	14.06	14.60	14.05
1st torsion	31.05	31.14	31.04
1st in-plane bending	31.72	31.73	31.71
3rd bending	39.36	44.01	39.35

The computed structural frequencies agree with the exact beam theory solutions to within 0.1% for all five modes at the production mesh, and correspond closely to the previously published data [2]; the largest discrepancy with the reference data occurs for the third bending mode, where Patil’s reported value exceeds the exact beam solution. The overall level of agreement provides confidence in the accuracy of the finite-element discretisation and the mass/stiffness assembly.

#### Static aeroelastic deflections

**Fig. 4:**
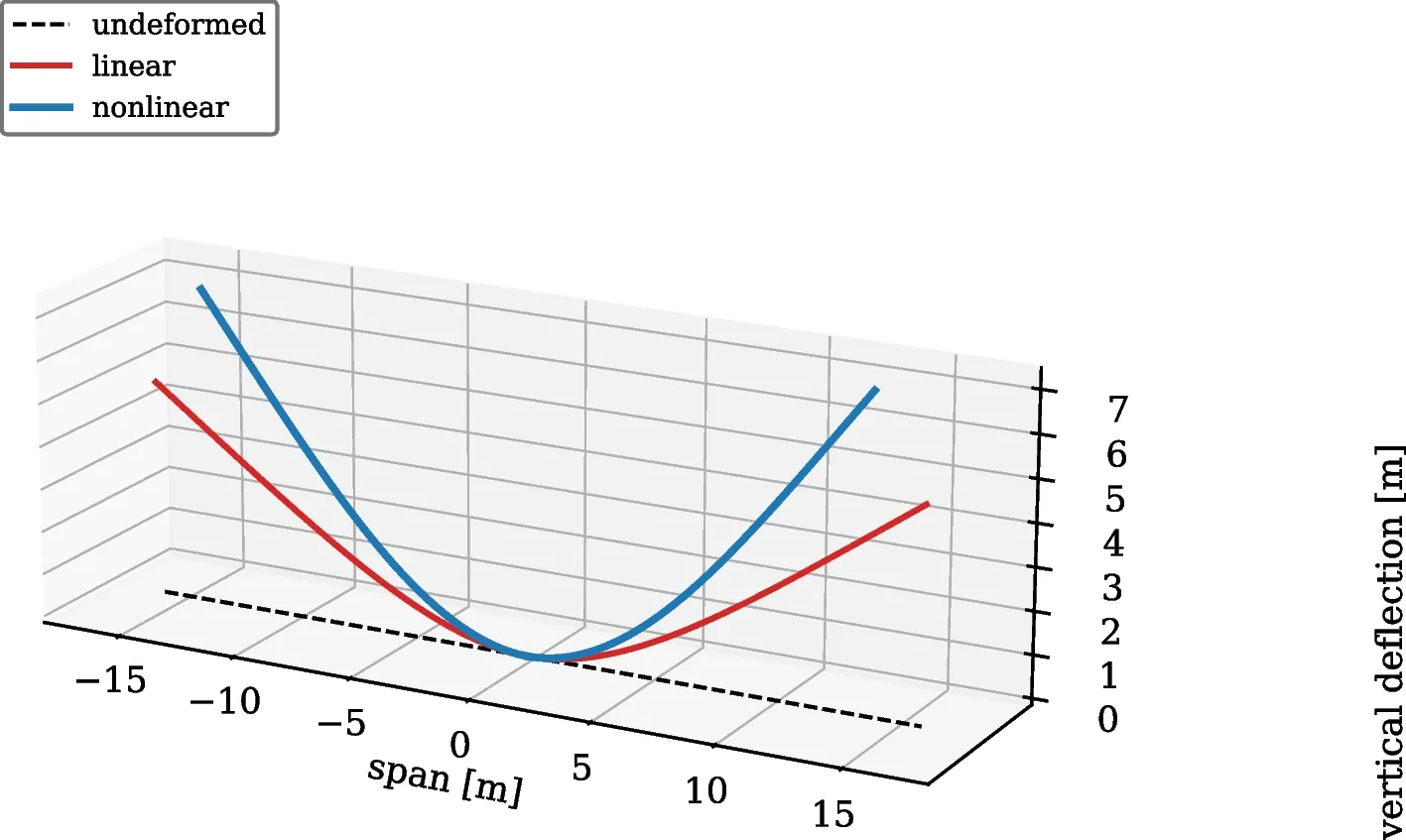
Three-dimensional deformed wing shapes: comparison of the linear analysis, the geometrically nonlinear analysis, and the undeformed configuration (*σ = 1*, $\alpha = 4^\circ$, *U = 25* m/s). The full span is shown by mirroring the computed semi-span; the two small squares at the origin mark the root chord. The chord axis (horizontal, *± 0.4* m) is illustrative and serves only to give the three-dimensional perspective

Figure 4 presents a three-dimensional comparison of the deformed wing shapes for the linear analysis, the geometrically nonlinear analysis, and the undeformed baseline configuration at *U = 25* m/s and *ρ = 0.0889* kg/m$^3$. Both analyses come from the present framework and differ only in their treatment of the deformation: the linear solution evaluates the loads once on the undeformed geometry and uses the fixed elastic stiffness $\boldsymbol{K}_e$, whereas the nonlinear solution converges the follower loads and the tangent stiffness on the deformed configuration.

Two opposing nonlinear mechanisms distinguish the solutions, and at this flight condition their net effect is that the linear analysis *underpredicts* the deflection substantially (tip deflection 4.99 m linear against 7.33 m nonlinear at $\alpha =4^\circ$). The dominant mechanism is the wash-in feedback between elastic twist and lift discussed above: the nose-up quarter-chord moment increases the local incidence, which increases the lift and hence the bending deformation, a loop that the one-shot linear solution cannot represent and that is strong at 45% of the divergence dynamic pressure. Acting against it, the geometric effects captured by the nonlinear kinematics—the rotation of the local lift vectors with the growing dihedral, the kinematic foreshortening of the planform (the tip moves inboard by 2.1 m, or 13% of the semi-span, at $\alpha =4^\circ$), and the stress stiffening represented by $\boldsymbol{K}_g$ in Eq. (9)—limit the deflection growth and are responsible for the visibly sub-parabolic shape of the nonlinear solution. Which side dominates is configuration- and condition-dependent: for torsionally stiff wings (or elastic axes near the aerodynamic centre) the wash-in loop is weak and linear analysis errs on the high side, whereas for the present benchmark the twist–lift coupling prevails. This underlines a central point of the paper: the sign of the linear-analysis error is not known a priori, and only the converged nonlinear solution establishes it. The progressive increase in effective dihedral with aerodynamic loading is also evident in Fig. 4, where the wing assumes a pronounced curved shape that significantly alters the direction in which each spanwise section generates lift.

#### Flutter speed

The flutter speed is obtained by tracking the eigenvalues of the linearised first-order coupled system (structure plus aerodynamic lag states) as the airspeed is increased, with the flutter point located by bisection on the sign change of the largest real part among the oscillatory modes; the linearisation is evaluated about the undeformed equilibrium for direct comparison with the published values in Table 5.

**Table 5:** Flutter speed (m/s) comparison for the HALE wing (*σ = 1*, undeformed equilibrium)

Method	Flutter speed (m/s)
Present (strip theory)	32.7
Patil and Hodges [2]	32.2
Murua et al. [11]	33.0

The flutter speed of 32.7 m/s predicted by the present framework lies within 1.5% of the Patil and Hodges value and within 1% of the UVLM-based prediction reported by Murua et al. The associated flutter frequency of 22.1 rad/s agrees closely with the 22.6 rad/s reported by Patil and Hodges. The flutter mechanism involves the coalescence of the torsion branch with out-of-plane bending, consistent with the classical binary description [2]. In addition, the torsional divergence pressure implied by the present aerodynamic and stiffness models corresponds to a divergence speed of 37.2 m/s, in exact agreement with the value reported by Patil and Hodges; this provides an independent check on the static twist–lift coupling. This level of agreement with independent reference solutions provides strong validation of the present coupled framework.

### Static wing deformation profiles

**Fig. 5:**
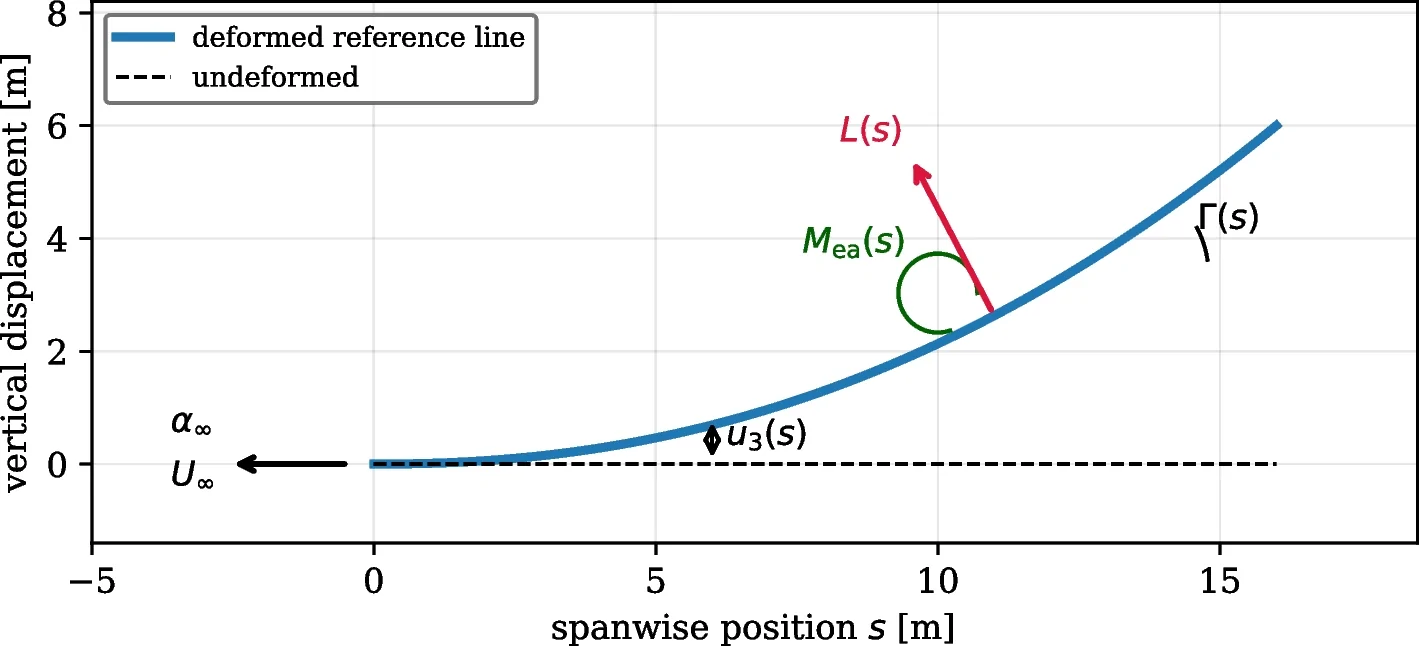
Schematic of the deformed wing showing the definitions of the sectional lift *L*(*s*), pitching moment $M_{\textrm{ea}}(s)$, vertical displacement $u_3(s)$, local dihedral angle (*Γ (s)*), angle of attack ($\alpha _\infty$), and freestream velocity ($U_\infty$)

Before proceeding to the full parametric study, it is instructive to illustrate the key kinematic quantities that govern the coupled aeroelastic–flight dynamic behaviour. Figure 5 presents a schematic of the deformed wing configuration, showing the definitions of lift force, pitching moment, vertical displacement, local dihedral angle, angle of attack, and freestream velocity used throughout the parametric study.

**Fig. 6:**
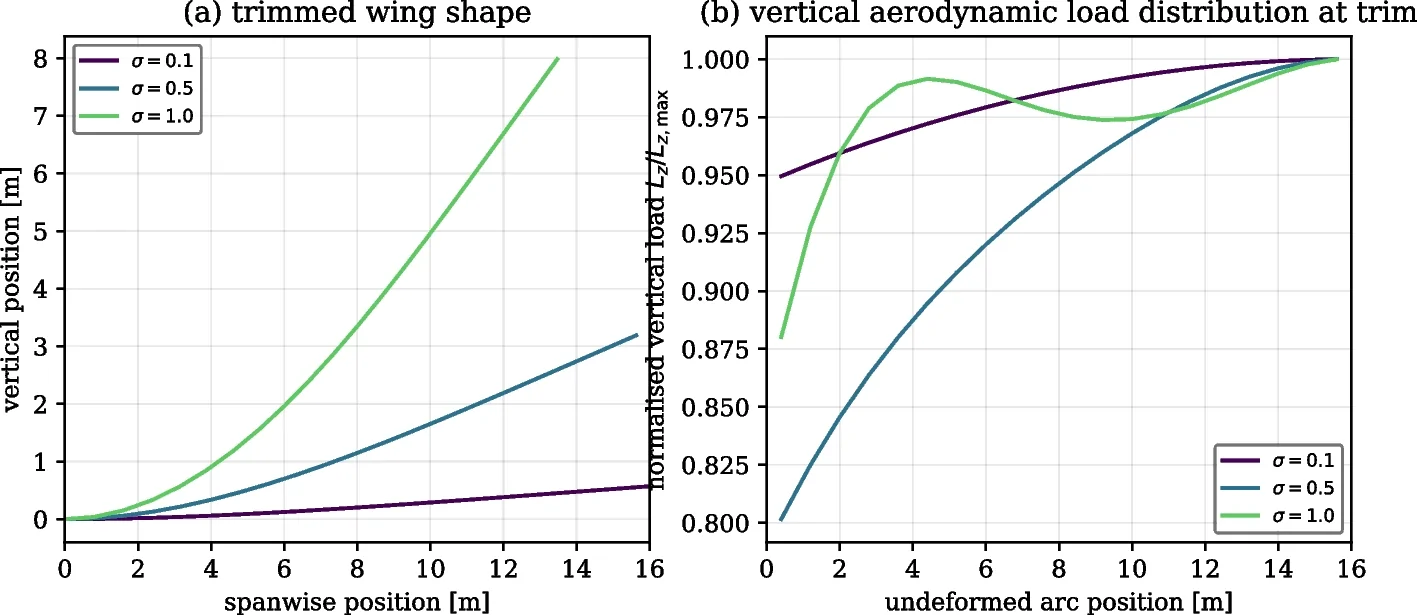
Trimmed equilibria across the flexibility range: **a** deformed wing shapes; **b** spanwise distribution of the vertical component of the aerodynamic load, normalised by its maximum (*U=25* m/s, right semi-span shown). At high flexibility the outboard sections feather out of the vertical force balance and the load concentrates inboard

The computed trimmed wing shapes and the corresponding spanwise distributions of vertical aerodynamic load are shown in Fig. 6 for three representative flexibility levels spanning the physically valid range. As the flexibility parameter *σ * increases, the static wing deformation grows smoothly and monotonically. For low flexibility ($\sigma \le 0.1$, trimmed tip deflection below 4% of the semi-span) the deformation follows the classical, nearly parabolic profile of linear beam theory, and the vertical load distribution is almost uniform along the span—the expected result for an untwisted, constant-chord wing under two-dimensional strip aerodynamics—with a mild outboard bias produced by the elastic wash-in ($0.4^\circ$ tip twist at *σ =0.1*). For moderate flexibility (*σ = 0.5*–1.0, tip deflections of 20–50% of the semi-span) the profile steepens progressively while the wash-in (elastic tip twist) grows to about $4.9^\circ$ at the tip, shifting the load maximum outboard; the deformation remains a smooth, monotonic bending state, but its amplitude is already far beyond the linear regime.

The range shown terminates at *σ = 1* (a tip deflection of half the semi-span) because the trimmed equilibrium remains physically valid only up to $\sigma \approx 1.25$. Beyond that point the required trim incidence exceeds the aerofoil stall angle (Trim and flutter speed variation with flexibility section), and for $\sigma \gtrsim 2.2$ the flight speed exceeds the linear torsional divergence speed. In that regime the attached-flow strip model formally continues to return equilibria—the wing folding progressively as the outboard sections feather out of the flow—but a real wing would stall and diverge, so these deeply deformed states are artefacts of the model applied outside its range of validity and are not presented as physical results. The valid range nonetheless already spans from an effectively rigid wing to a tip deflection of half the semi-span, comfortably covering the flexibility of current and near-term HALE designs.

Even within the valid range this behaviour cannot be captured by superposition of linear mode shapes: the elastic wash-in redistributes the load, shifting its maximum outboard as flexibility increases, which is directly relevant to structural sizing. The growing mean geometric dihedral of the deformed wing likewise carries implications for the flight dynamics, as discussed in the subsequent sections.

### Trim analysis: flexible vs. rigid

The trim analysis determines the equilibrium angle of attack $\alpha _{\textrm{trim}}$ and thrust $T_{\textrm{trim}}$ that simultaneously satisfy force and pitching-moment equilibrium at a prescribed flight speed and altitude, following the procedure detailed in Nonlinear trim procedure section. For the flexible aircraft, this is an inherently nonlinear problem: the wing deformation depends on the aerodynamic loads, which themselves depend on the deformation, creating a mutual dependence that is resolved by the nested Newton iteration of Nonlinear trim procedure section, in which the full nonlinear static aeroelastic problem is converged at every trim iterate.

**Fig. 7:**
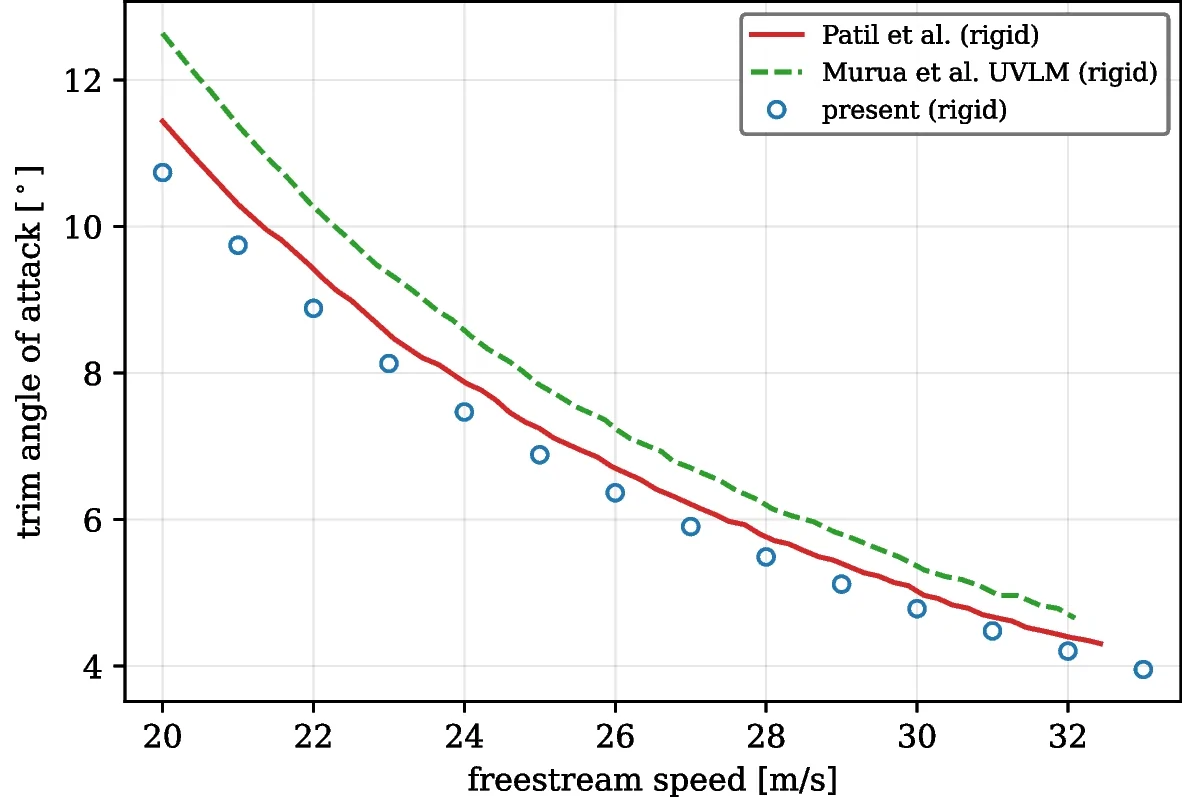
Trim angle of attack versus freestream speed for the rigid aircraft: comparison of the present strip-theory model (symbols) with Patil et al. [2] (solid line) and Murua et al. [11] UVLM (dashed line)

Figure 7 shows the trim angle of attack as a function of freestream speed for the rigid aircraft, comparing the present strip-theory predictions with the results of Patil et al. [2] and the UVLM-based predictions of Murua et al. [11].

The present results are in good agreement with both reference datasets across the speed range, tracking the Patil et al. curve with a small offset that decreases from about $0.7^\circ$ at the lowest speed to about $0.2^\circ$ near *U=31* m/s, attributable to the idealised quasi-steady tail model; the UVLM predictions sit systematically higher because the three-dimensional aerodynamics generate less lift for the same incidence. The trim angle of attack decreases with increasing speed as expected from the increase in dynamic pressure.

The influence of *flexibility* on trim is governed by two competing mechanisms, and it is essential to recognise that they act in opposite directions. The first is the elastic wash-in produced by the nose-up quarter-chord moment about the mid-chord elastic axis: torsional flexibility raises the local incidence along the span, so the flexible wing generates *more* lift than the rigid wing at the same attitude, and the trim angle initially *falls* as flexibility increases. The second is the lift vector rotation effect. When the wing deflects upward under aerodynamic loading, the local lift vectors at each spanwise station rotate inward, producing a net vertical lift component that falls short of the total aerodynamic force magnitude. To make the second mechanism quantitative, consider a wing element at spanwise location *s* with a local dihedral angle *Γ (s)* arising from bending deformation. The vertical component of the local lift is $L(s)\cos \Gamma (s)$, while the lateral (inboard-directed) component is $L(s)\sin \Gamma (s)$. The total vertical force is accordingly

40 $$\begin{aligned} F_z = \int _0^L L(s)\cos \Gamma (s)\,\textrm{d}s < \int _0^L L(s)\,\textrm{d}s, \end{aligned}$$

since $\cos \Gamma (s) < 1$ for any non-zero dihedral angle. In essence, the wing deformation generates an effective dihedral that redirects a portion of the lift force laterally rather than vertically; to restore vertical force balance ($F_z = W$), the angle of attack must be increased to generate additional total lift. This mechanism was first identified and described by Patil et al. [2]. Because the wash-in scales with the twist (roughly linearly in the deformation) while the dihedral deficit grows quadratically, Eq. (42), the wash-in relief dominates at moderate flexibility and the lift-rotation penalty dominates at high flexibility: the trim angle as a function of *σ * is therefore non-monotonic, first decreasing and then rising steeply, as quantified in Trim and flutter speed variation with flexibility section.

As the flexibility parameter *σ * grows, the wing-tip deflection at trim increases in a strongly nonlinear fashion—from 7% of the semi-span at *σ =0.2* to 42% at *σ =0.9* in the present computations—eventually reaching large fractions of the semi-span at the highest flexibility levels. The behaviour of the required trim angle over this range reflects the competition described above, and its high-flexibility branch, where $\alpha _{\textrm{trim}}$ rises with growing steepness as the $\cos \Gamma$ deficit compounds, has direct implications for the available angle-of-attack margin before stall, which shrinks as the aircraft becomes more flexible.

### Trim and flutter speed variation with flexibility

**Fig. 8:**
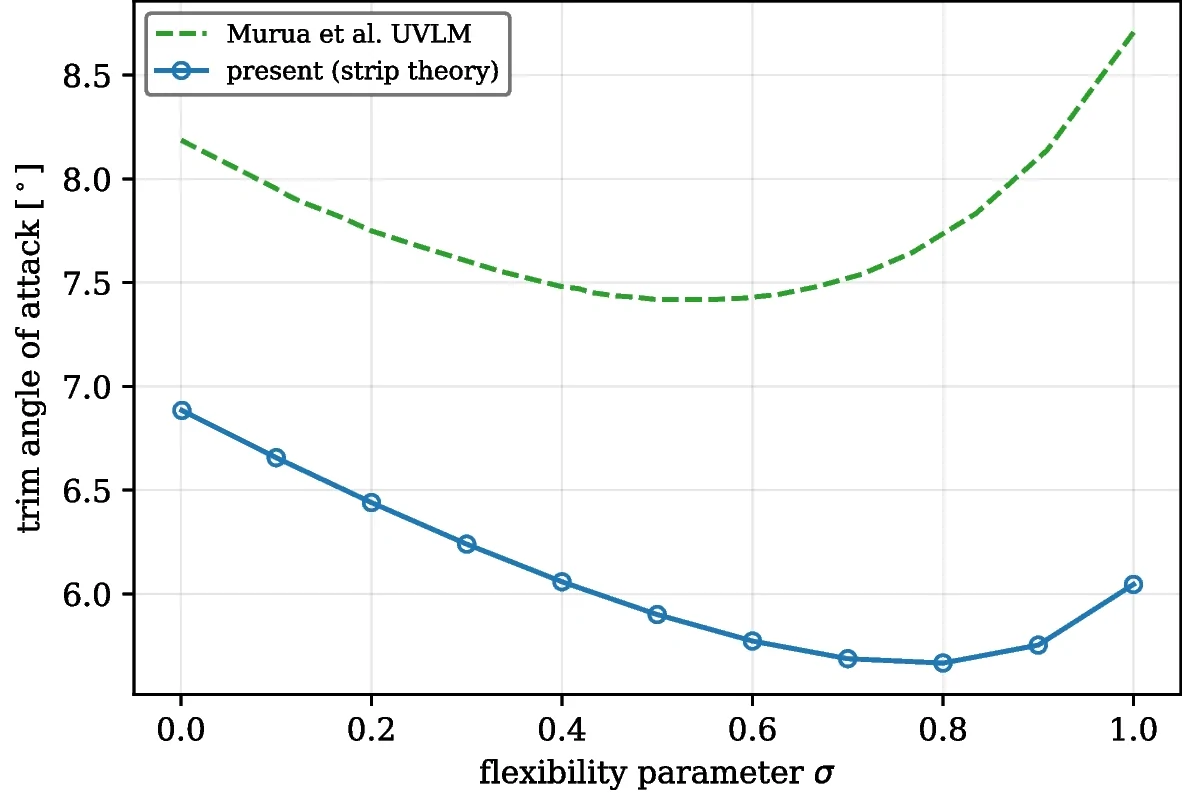
Trim angle of attack versus flexibility parameter *σ *: comparison between the present strip-theory model and the UVLM-based results of Murua et al. [11]

A question of central importance for the design of very flexible aircraft concerns how the trim conditions change with the level of structural flexibility. Figure 8 presents the trim angle of attack as a function of the flexibility parameter *σ *, comparing the present strip-theory predictions with the UVLM-based results of Murua et al. [11].

The trim angle of attack is *non-monotonic* in *σ *, and the same characteristic shape is exhibited by both aerodynamic models: starting from the rigid value, $\alpha _{\textrm{trim}}$ first decreases as torsional wash-in raises the lift available at a given attitude, passes through a shallow minimum, and then rises with increasing steepness as the bending-induced dihedral redirects a growing share of the lift laterally. In the present computations the minimum occurs near $\sigma \approx 0.8$, where the trim angle is about $1.2^\circ$ below the rigid value; the UVLM reference of Murua et al. displays the same dip-and-rise shape with a shallower minimum near $\sigma \approx 0.5$, the quantitative differences being consistent with the stronger wash-in of the two-dimensional strip aerodynamics already identified in the static validation (HALE aircraft validation section). The agreement in the shape of the curve—produced by two independently formulated aerodynamic models—supports the two-mechanism interpretation advanced in Trim analysis: flexible vs. rigid section. Beyond the valid range the trim angle rises steeply and reaches the aerofoil stall angle near $\sigma \approx 1.35$ ($9.7^\circ$ at *σ = 1.25*, $14.9^\circ$ at *σ = 1.5*); moreover, because the uniform stiffness scaling reduces *GJ* along with the bending stiffnesses, the linear torsional divergence speed $U_D = 37.15/\sqrt{\sigma }$ m/s falls below the 25 m/s flight speed for $\sigma \gtrsim 2.2$. The trimmed states at higher flexibility are therefore both stalled and, further on, super-divergent; although the attached-flow strip model still returns equilibria there (the outboard sections feathering out of the flow), those states lie outside the model’s range of validity and are not interpreted as flyable conditions, a point taken up in the Limitations section and reflected in the criteria of Table 6. The valid range, $\sigma \lesssim 1.25$, already covers tip deflections up to 72% of the semi-span.

**Table 6:** Analysis-fidelity criteria derived from the parametric study (*U=25* m/s, *h=20* km). $\delta _{\textrm{tip}}/L$ is the trimmed tip-deflection ratio computed by the present model

Regime	Range	Characteristic behaviour	Required analysis
I: quasi-linear	$\sigma \lesssim 0.1$ ($\delta _{\textrm{tip}}/L \lesssim 0.04$)	Trim within $0.25^\circ$ of rigid; wash-in twist $<0.5^\circ$; flutter and divergence far outside envelope; no unstable slow mode.	Linear static aeroelasticity and conventional rigid-body flight dynamics; decoupled autopilot design acceptable.
II: moderately flexible	$0.1 \lesssim \sigma \lesssim 1.25$ ($0.04 \lesssim \delta _{\textrm{tip}}/L \lesssim 0.72$)	Non-monotonic trim (wash-in dip of $1.2^\circ$, then rise); pre-stress correction to flutter $\lesssim 3.5\%$; longitudinal flight-dynamic (phugoid) stability eroded, active control required [5]; direct gust bending loads alleviated across the span (peak amplification *2.8 → 1.7*–1.9), but the uncontrolled response does not self-arrest.	Nonlinear static (trim) analysis for loads and trim drag; linearised stability about the *deformed* trim state; active stability augmentation mandatory; gust loads from coupled simulation with the full spanwise envelope.
III: beyond validity	$\sigma \gtrsim 1.25$ ($\delta _{\textrm{tip}}/L \gtrsim 0.72$)	Trim incidence exceeds aerofoil stall ($\alpha > 12^\circ$ for $\sigma \gtrsim 1.35$) and the flight speed exceeds the linear divergence speed for $\sigma \gtrsim 2.2$; the attached-flow model still returns folded, feathered equilibria but they are not flyable. The undeformed (linear) flutter analysis remains valid and places the flutter speed below cruise for $\sigma \gtrsim 1.75$.	The present strip-theory/attached-flow model is outside its validity; a higher-fidelity model (stall-capable, three-dimensional/viscous aerodynamics, with a proper flight-dynamics and control model) is required. In practice this regime signals that the configuration should be stiffened back into regime II rather than analysed as-is.

**Fig. 9:**
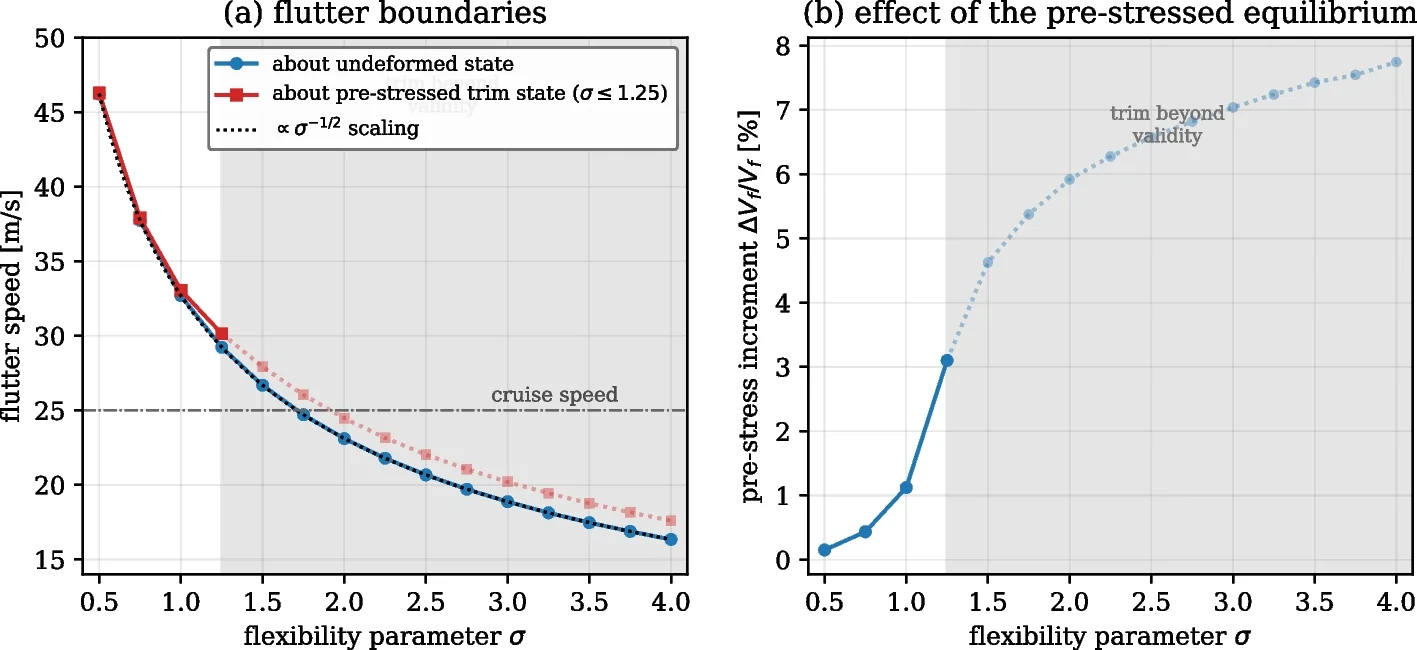
Effect of flexibility on the flutter boundary. **a** Flutter speed versus flexibility parameter *σ *, computed from linearisation about the undeformed configuration (valid at any stiffness) and about the pre-stressed trimmed equilibrium (deformation frozen at the 25 m/s cruise trim state), with the classical $\sigma ^{-1/2}$ scaling and the 25 m/s cruise speed shown for reference. **b** The pre-stress increment $\Delta V_f/V_f$ (pre-stressed minus undeformed, relative to undeformed). The pre-stressed curve is drawn solid where the trimmed equilibrium is physically valid ($\sigma \le 1.25$) and dotted in the shaded region beyond, where the trim state is stalled/super-divergent and outside model validity; within the valid range the pre-stress increment reaches about 3%

The flutter boundary is computed in two ways for every value of *σ *, and the comparison—requested by the reviewers and shown in Fig. 9—quantifies the role of the equilibrium state directly. In the first analysis the coupled system is linearised about the undeformed configuration; in the second it is linearised about the pre-stressed trimmed equilibrium, with the deformation frozen at the 25 m/s cruise trim state so that the pre-stress effect is isolated at the operating point. When the analysis is performed about the undeformed equilibrium, the computed flutter speed follows the classical scaling $V_f \propto \sigma ^{-1/2}$ essentially exactly (32.68 m/s at *σ =1*, 23.11 at *σ =2*, 16.34 at *σ =4*, against scaling predictions of 23.11 and 16.34 m/s respectively), as it must, since the uniform stiffness scaling leaves the mode shapes of the undeformed wing unchanged.

The pre-stressed analysis, over the range in which the trimmed equilibrium is physically valid ($\sigma \lesssim 1.25$), predicts a slightly higher flutter speed than the undeformed analysis, the difference growing with flexibility: *+0.2%* at *σ =0.5*, *+1.1%* at *σ =1*, and about *+3%* at *σ =1.25*. The origin is the geometric (stress) stiffening of the deformed wing, which raises the effective stiffness-to-aerodynamic-loading ratio of the configuration about which the perturbation dynamics evolve. The practical message is that conventional flutter analysis about the undeformed geometry is mildly conservative for flexible configurations—by up to a few percent within the valid range—so that basing flutter clearance on the correctly computed deformed equilibrium recovers a small but non-negligible margin; beyond the valid range the pre-stressed analysis would predict larger increments, but these are evaluated about the stalled, super-divergent trim states discussed above and are not reported as predictions.

Whichever equilibrium is used, the flutter speed itself falls steeply with flexibility: the undeformed scaling gives 32.7 m/s at *σ = 1* and drops below the 25 m/s cruise speed for $\sigma \gtrsim 1.75$. A wing this flexible would therefore encounter flutter before even reaching its cruise condition—an entirely valid conclusion of the linear (undeformed) analysis, which requires no flyable trimmed state—underlining that flutter, rather than any static-aeroelastic subtlety, is the binding constraint at high flexibility.

**Fig. 10:**
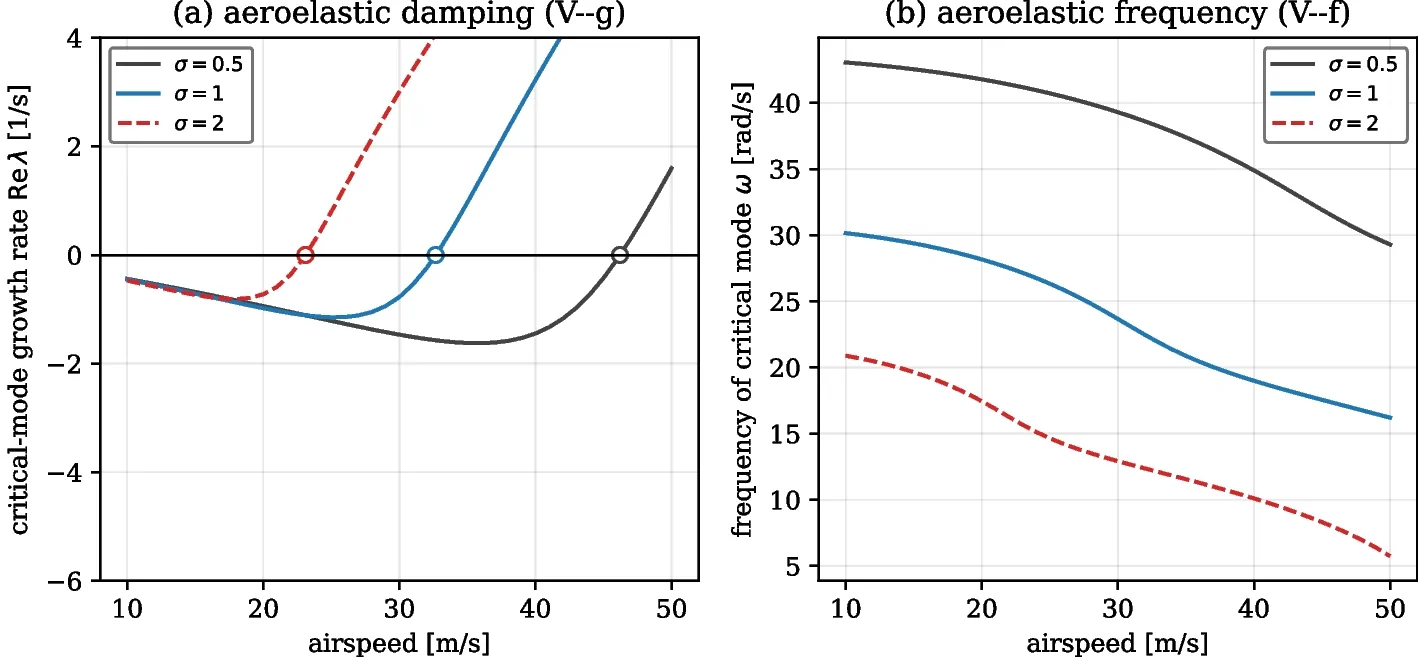
Aeroelastic mode tracking: growth rate (**a**) and frequency (**b**) of the critical aeroelastic mode versus airspeed for *σ =0.5*, 1, and 2, from the clamped-wing analysis. Open circles mark the flutter crossing (damping through zero), which moves to lower speed and lower frequency with increasing flexibility. The longitudinal flight-dynamic (phugoid, short-period) eigenvalues are deliberately not shown here; see the discussion at the end of this subsection and Limitations section

The character of the flutter mechanism itself also evolves with flexibility, as made explicit by the aeroelastic mode-tracking diagram of Fig. 10, discussed in the next subsection. The flutter instability arises through the coalescence of a bending branch with the torsion branch, consistent with the classical binary flutter problem; as flexibility increases, the coalescence occurs at a lower airspeed and a lower frequency (the critical frequency falls from about 22 rad/s at *σ =1* to about 16 rad/s at *σ =2*), reflecting the downward shift of the whole aeroelastic spectrum with reducing stiffness.

### Flexibility effect on flight dynamic response

Attention now turns to the dynamic behaviour of the coupled system about the trimmed equilibrium, distinguishing—as requested in review—the aeroelastic modes, which the present framework resolves reliably, from the longitudinal flight-dynamic modes, which its simplified free-flight representation does not (as detailed at the end of this subsection and in Limitations section).

The aeroelastic modes are tracked across airspeed and flexibility in Fig. 10, which presents the growth rate (panel a) and frequency (panel b) of the critical aeroelastic mode as functions of airspeed for three flexibility levels, obtained from the validated clamped-wing analysis of Flutter speed section. The mode is heavily damped at low speed, and its damping decreases as the dynamic pressure rises until it crosses zero at the flutter speed—the open circles—which marches from about 46 m/s at *σ = 0.5* to 32.7 m/s at *σ = 1* and 23.1 m/s at *σ = 2*, in exact agreement with the flutter boundary of Fig. 9. Panel (b) shows the critical frequency at the crossing decreasing with flexibility—from about 31 rad/s at *σ = 0.5* to 22 rad/s at *σ = 1* and 16 rad/s at *σ = 2*: the flutter mechanism remains a bending–torsion coalescence throughout, but shifts bodily to lower frequency as the stiffness is reduced. This mode-tracking view makes the flexibility dependence of the flutter mechanism explicit and provides the aeroelastic-mode data requested by the reviewers.

The dynamic gust response of the coupled system, and the load alleviation that flexibility provides, are examined in detail in Gust response: rigid vs. flexible section.

The longitudinal flight-dynamic modes—the short-period and, especially, the phugoid—require a separate and candid comment. Patil and Hodges [5] established, with a dedicated nonlinear flight-dynamics model, that wing flexibility destabilises the phugoid of very flexible aircraft, a result of central importance for control system design. The framework used here was constructed and validated for the *aeroelastic* response—natural frequencies, flutter, divergence, static deformation, and gust bending loads—and its longitudinal flight-dynamic representation is deliberately simplified: the pitch attitude is carried by a root feathering mode rather than an independent rigid-body rotation, the thrust is held constant, and the controls are fixed. This representation does not reproduce the textbook rigid-aircraft phugoid quantitatively, as we verify and discuss in Limitations section. We therefore do not report longitudinal flight-dynamic eigenvalues as a quantitative result. The qualitative mechanism by which flexibility acts on the phugoid is nonetheless well understood and physically robust: as the wing bends upward, the effective aerodynamic centre of the deformed wing migrates relative to the centre of gravity, altering the pitch restoring moment, and the effective dihedral diminishes the vertical-force lift-curve slope—only the $\cos \Gamma$ component of each strip’s lift supports the weight—which reduces the aerodynamic damping of the speed–height exchange. Our results are consistent with the destabilising trend reported by Patil and Hodges, but its quantification for a specific configuration requires a dedicated flight-dynamics model and is left to future work.

The substantial static deformation at high values of *σ * also gives rise to an effective geometric dihedral angle $\Gamma _{\textrm{eff}}$. This effective dihedral introduces lateral-directional coupling that is absent in the rigid, planar-wing model, indicating that the longitudinal and lateral dynamics cannot in general be treated separately for very flexible configurations—a coupling that a full six-degree-of-freedom flight-dynamic study, beyond the present longitudinal scope, would need to address.

### Effect of lift vector rotation on flight dynamics

The lift vector rotation mechanism warrants further quantitative examination, as it constitutes the primary driver of the flexibility-induced modifications to both trim and stability. For a wing element located at spanwise position *s* with local vertical displacement $u_3(s)$ and slope $\textrm{d}u_3/\textrm{d}s = \tan \Gamma (s)$, the lift vector resolved in the inertial frame possesses components:

41 $$\begin{aligned} \boldsymbol{L}_{\textrm{inertial}}(s) = L(s) \begin{bmatrix} -\sin \alpha _{\textrm{eff}} \\ -\sin \Gamma \cos \alpha _{\textrm{eff}} \\ \cos \Gamma \cos \alpha _{\textrm{eff}} \end{bmatrix}, \end{aligned}$$

where the first component acts in the flight direction (contributing to induced drag), the second is lateral (generating a side force), and the third is vertical (supporting the aircraft weight). The vertical force deficit relative to a rigid wing can be quantified as

42 $$\begin{aligned} \Delta F_z = \int _0^L L(s)\left[ 1 - \cos \Gamma (s)\right] \textrm{d}s \approx \int _0^L \frac{L(s)\Gamma ^2(s)}{2}\,\textrm{d}s, \end{aligned}$$

where the approximate equality holds for moderate dihedral angles and is obtained via a Taylor expansion of the cosine function. Since the dihedral angle *Γ (s)* increases with *σ *, the vertical force deficit grows quadratically with the deformation level. This quadratic dependence explains both why the wash-in relief (linear in the deformation) prevails at moderate flexibility and why the lift-rotation penalty overtakes it at high flexibility, producing the accelerating growth of $\alpha _{\textrm{trim}}$ along the high-*σ * branch of the trim curve (Trim and flutter speed variation with flexibility section).

The lateral force component generated by the lift vector rotation is

43 $$\begin{aligned} F_y = \int _0^L L(s)\sin \Gamma (s)\,\textrm{d}s, \end{aligned}$$

which, for the symmetric configuration studied here, vanishes in the trimmed state because the contributions from the left and right semi-spans cancel exactly. During asymmetric perturbations, however—such as those caused by a lateral gust or a roll disturbance—the lateral force becomes non-zero and functions as a restoring force analogous to the dihedral effect in conventional aircraft design. The effective dihedral derivative $C_{l_\beta }$ increases with *σ *, which strengthens the Dutch roll stability but may simultaneously introduce excessive roll damping that could compromise manoeuvrability. This trade-off between stability and handling qualities must be carefully managed in the design of very flexible aircraft.

### Gust response: rigid vs. flexible

The dynamic response to atmospheric gusts is explored using a “1-minus-cosine” vertical gust profile of the form:

44 $$\begin{aligned} w_g(t) = \frac{w_{g0}}{2}\left( 1 - \cos \frac{2\pi U t}{H_g}\right) , \quad 0 \le t \le \frac{H_g}{U}, \end{aligned}$$

where $w_{g0} = 5$ m/s is the peak gust velocity and $H_g = 25c = 25$ m is the gust length (so that the gust gradient distance, i.e. the distance to the peak, is $H_g/2 = 12.5$ m). The gust is applied uniformly across the span (simultaneous penetration) for the initial comparison, representing a spatially coherent disturbance. The gust velocity ratio $w_{g0}/U = 0.2$ corresponds to a moderately severe gust encounter under typical HALE operating conditions.

**Fig. 11:**
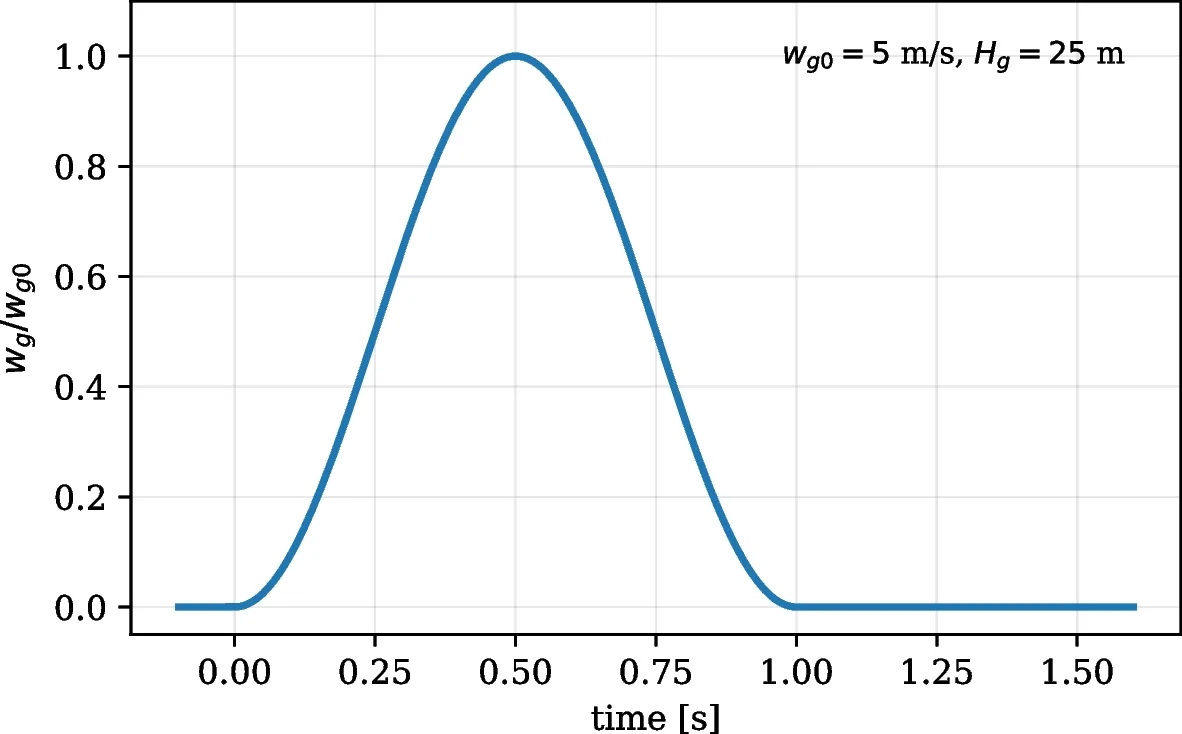
Normalised 1-minus-cosine gust velocity profile ($w_{g0} = 5$ m/s, $H_g = 25$ m)

**Fig. 12:**
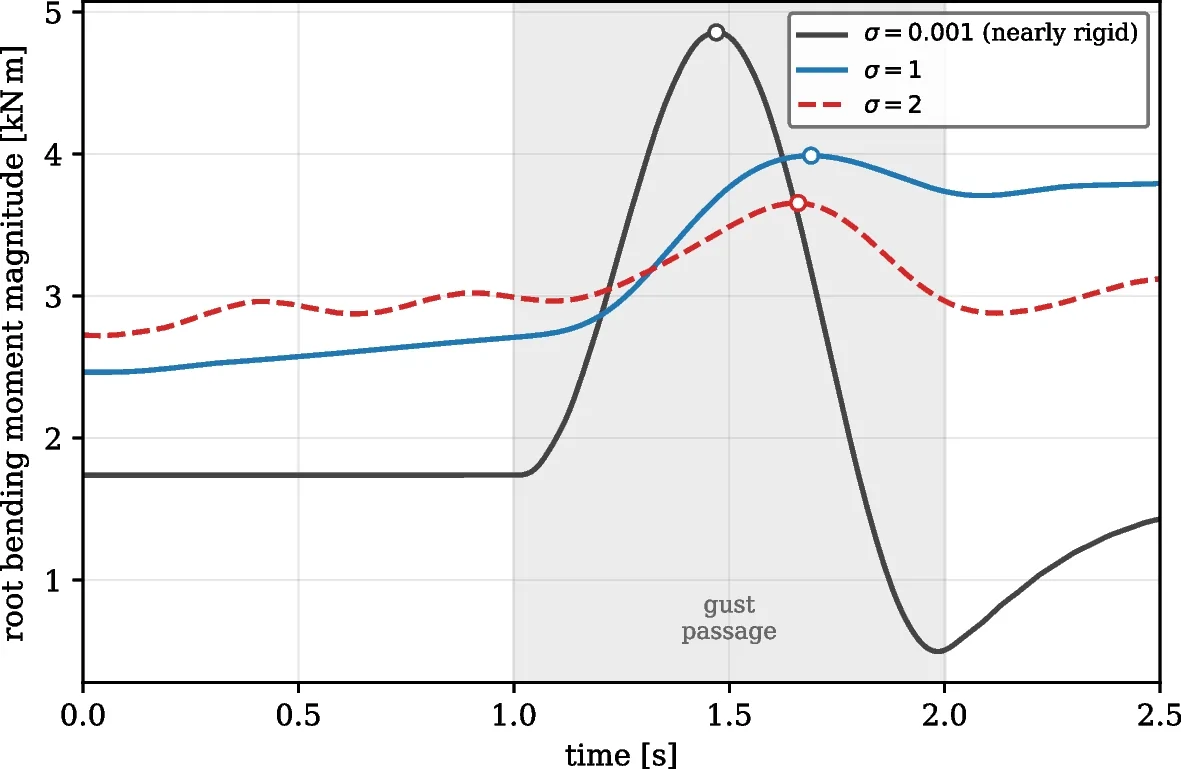
Root bending-moment magnitude during the gust passage for *σ = 0.001* (nearly rigid), *σ = 1*, and *σ = 2*; open circles mark the peaks and the shaded band is the gust passage (*t∈ [1,2]* s). Although the flexible configurations carry a higher trim moment, their *absolute* peak gust load is lower than that of the rigid wing—4.0 and 3.7 kN m at *σ = 1* and 2 against 4.9 kN m at *σ = 0.001*—demonstrating the load-alleviation benefit of flexibility. The window is limited to the physically valid gust-passage response; the subsequent free-flight longitudinal motion is not resolved reliably by the present model (Limitations section) and is not shown

Figure 11 shows the normalised gust velocity profile used in the simulations, and Fig. 12 presents the resulting root bending-moment time history over the gust passage for the near-rigid, nominal, and moderately flexible configurations.

For the nearly rigid configuration (*σ = 0.001*), the wing-tip deflection remains at the centimetre level and the root bending moment exhibits a clean, quasi-steady response that closely mirrors the temporal profile of the gust input (Fig. 12): the computed peak occurs at *t = 1.47* s, essentially coincident with the peak of the gust input, and reaches 2.79 times the trim value (4.9 kN m against a 1.7 kN m trim moment), in close agreement with the quasi-steady estimate based on the instantaneous gust incidence increment. After the gust passes, the loads decay essentially back to their trim values and the structural dynamics play no significant role.

For the moderately flexible configuration (*σ = 1*), the direct response during the gust passage demonstrates the classical load-alleviation benefit of flexibility, now quantified (Fig. 12): the root bending moment rises to only 1.62 times its trim value (against 2.79 for the rigid wing), and, despite the flexible wing carrying a 42% higher trim root moment, its absolute peak moment during the gust passage is 18% *lower* than that of the rigid wing (4.0 kN m against 4.9 kN m). As the wing bends upward under the gust load, the local incidence and the vertical lift component both fall, shedding load exactly as the alleviation argument predicts. Following the gust, the response does not simply decay: the coupled pitch–plunge–bending motion develops a growing longitudinal excursion, consistent with the marginal longitudinal stability of this class of configuration. As set out in Flexibility effect on flight dynamic response section, the quantitative growth rate of this longitudinal motion is governed by the free-flight dynamics that the present simplified model does not resolve reliably; the robust and design-relevant result here is the direct-response alleviation during the gust passage, together with the qualitative fact that the uncontrolled response of the flexible aircraft does not self-arrest and therefore mandates active stability augmentation.

The spanwise structure of the dynamic loads during the gust passage confirms that the alleviation acts along the entire span, not merely at the root. Normalising the peak bending-moment envelope over the gust passage by the trim distribution, the rigid wing shows an almost uniform amplification of about 2.8 along the span, whereas at *σ = 1* the amplification is no greater than about 1.9 anywhere along the span (roughly 1.6–1.7 at the root, rising mildly to 1.9 outboard): the flexible wing sheds load everywhere during the gust, the alleviation being only marginally less effective at the outboard stations. There is thus no adverse dynamic-load amplification relative to the rigid wing during the direct gust response. The much larger, tip-concentrated excursions that develop at longer times are governed by the free-flight longitudinal dynamics discussed in Flexibility effect on flight dynamic response section and are not part of the reliable direct-gust loading.

At *σ = 2* the amplification during the gust passage falls further, to 1.34 (Fig. 12), continuing the alleviation trend; it should be borne in mind, however, that the *σ = 2* trimmed state already lies in the beyond-validity regime of the model (trim incidence of order $20^\circ$, past aerofoil stall, and close to the linear divergence boundary), so this value is indicative of the trend rather than a certified load. Beyond this point the model can no longer furnish meaningful gust loads: at *σ = 4* the trimmed wing is folded and stalled, and the gust drives the coupled response into departure within the gust passage itself (Limitations section). The physically grounded result of this section is therefore the load alleviation established across the valid flexibility range—the peak gust bending moment falling, in absolute terms, from 4.9 kN m for the rigid wing to 4.0 kN m at the nominal *σ = 1*—with the trend continuing, but the model losing validity, at higher flexibility.

A practical consequence deserves emphasis. Following the gust, the uncontrolled response of the flexible aircraft does not settle back to trim but develops a growing longitudinal excursion, so that active stability augmentation is mandatory to keep the aircraft in controlled flight. The quantitative growth of that excursion is governed by the free-flight longitudinal dynamics, which the present simplified model does not resolve reliably (Limitations section), and by aerodynamics that eventually leave the attached-flow range of validity; the excursion is therefore to be read as a qualitative indication that the uncontrolled response does not self-arrest, not as a quantitative prediction. The direct gust-passage response and its load alleviation—the design-relevant results of this section—are free of these caveats.

## Discussion

The parametric study presented in this work uncovers several findings of direct practical relevance for the design of HALE and very flexible aircraft. These findings are discussed in turn below.

### When is linear analysis acceptable?

For low values of *σ *, which correspond to small wing-tip deflections relative to the semi-span, the discrepancies between linear and nonlinear predictions remain modest and are unlikely to affect design decisions. In this regime, standard linear aeroelastic tools continue to provide adequate accuracy for preliminary design purposes, provided that the trim state is correctly computed as the starting point for subsequent stability and loads analyses. For higher values of *σ *, however, the nonlinear effects become increasingly pronounced and a geometrically-exact structural formulation is strongly recommended to avoid potentially non-conservative predictions [42]. The transition between these regimes is gradual rather than abrupt, but the present results provide quantitative markers for identifying when the linear approximation breaks down.

### Phugoid destabilisation.

Structural flexibility erodes longitudinal flight-dynamic stability—the phugoid destabilisation established for this class of aircraft by Patil and Hodges [5]—with important ramifications for flight control system design. The present study reports this trend qualitatively rather than quantitatively: as explained in Flexibility effect on flight dynamic response and Limitations sections, the simplified longitudinal free-flight representation used here is not a substitute for a dedicated flight-mechanics model, and the paper does not attach eigenvalue-level numbers to the phugoid. What the coupled gust simulations do show robustly is that the uncontrolled response of the flexible aircraft to a routine disturbance does not self-arrest, so that active control intervention becomes necessary to ensure stable flight. The controller design must explicitly account for the coupling between structural and flight dynamic modes, as conventional decoupled autopilots—which treat the structural and flight dynamic subsystems independently—may prove inadequate for this task [5, 42]. A variety of active control strategies have been developed to address this challenge, including adaptive, model-predictive, and robust control schemes for gust load alleviation [29, 32, 33], as well as nonlinear controllers for flutter suppression that have been validated through both simulation and wind tunnel testing [30, 31]. Reduced-order models suitable for real-time implementation have also been developed [3, 16, 22], overcoming the computational cost barrier that would otherwise preclude model-based control of flexible aircraft in practice.

### Flutter speed degradation and the role of pre-stress.

The flutter speed analysis reveals a noteworthy subtlety: the flutter boundary is governed not solely by the structural stiffness but also by the equilibrium state about which the stability analysis is performed. Conventional flutter analysis based on the undeformed geometry yields conservative predictions for flexible configurations because it overlooks the geometric stiffening effect conferred by the pre-stress. At elevated flexibility levels, the difference between the flutter speeds predicted by the undeformed and pre-stressed analyses becomes significant and cannot be ignored. This finding indicates that for very flexible aircraft, flutter clearance studies should be grounded in analysis performed about the correctly-computed nonlinear equilibrium rather than the undeformed state. Furthermore, at higher flexibility levels, the flutter mechanism may change in character as the spacing between adjacent modal frequencies diminishes, which can complicate flutter analysis approaches that rely on tracking a single pair of coalescing eigenvalues.

### Gust load alleviation and its limits.

Flexibility provides beneficial alleviation of the gust-passage bending loads along the entire span, not merely at the root: the peak bending-moment amplification over the gust falls from an almost uniform 2.8 for the rigid wing to 1.7–1.9 at *σ =1* and 1.3–1.6 at *σ =2*, with only a mild outboard bias (the alleviation is marginally less effective at the tip than at the root). The direct-response benefit is therefore genuine and spanwise-distributed. Two qualifications matter for design, however. First, the benefit is non-monotonic in flexibility: once the wing folds and its outboard sections feather (Static wing deformation profiles section), the geometric capacity to shed load by bending is exhausted, and at *σ =4* the direct gust amplification rises again to 6.2. Second, the load *maximum over a complete gust event* is not in general set by the direct response but by the subsequent longitudinal motion; quantifying that motion requires the dedicated flight-dynamics model discussed in Limitations section, so the total achievable load benefit of flexibility is inseparable from the performance of the stability-augmentation system. The geometric stiffening of the deformed wing moderates the growth of deformation increments, but it is configuration-dependent and should not be relied upon as a universal protection mechanism without case-specific verification.

### Comparison with higher-fidelity aerodynamics.

The comparison with three-dimensional aerodynamic references splits cleanly by the character of the quantity. The dynamic and integral quantities are predicted closely: the flutter speed (32.7 m/s) falls between the Patil–Hodges value (32.2 m/s) and the UVLM-based prediction of Murua et al. (33.0 m/s), and the rigid-aircraft trim curve tracks the references over the full speed range. The static aeroelastic deflections, in contrast, are systematically overpredicted (HALE aircraft validation section) because two-dimensional strips provide neither tip relief nor induced-incidence reduction, and the resulting excess lift is amplified through the twist–lift feedback loop at this near-divergence condition. For structural loads the strip-theory predictions are therefore conservative, which is acceptable—though potentially costly—in preliminary sizing; for detailed design and airworthiness certification purposes, coupled CFD–CSD analysis is recommended [19, 20]. The on-the-fly generation of aerodynamic look-up tables from CFD simulations [43] offers a promising intermediate-fidelity strategy that captures three-dimensional and viscous aerodynamic effects while preserving sufficient computational efficiency for parametric investigations.

### Effective dihedral and lateral-directional coupling.

The substantial static deformation that develops at high flexibility ratios produces an effective dihedral that couples the longitudinal and lateral-directional dynamics of the aircraft. This coupling is not represented in conventional flight dynamic models that assume a symmetric, undeformed planform, and its omission can lead to erroneous stability predictions. For highly flexible configurations, a full six-degree-of-freedom coupled analysis is therefore essential, and the lateral-directional stability characteristics must be evaluated in conjunction with the longitudinal ones [9, 14]. The effective dihedral derivative $C_{l_\beta }$ grows with flexibility, which may create issues with the Dutch roll mode dynamics and roll handling qualities. Active flutter suppression strategies [31] may likewise need to incorporate the lateral-directional coupling when applied to very flexible configurations.

### Implications for HALE aircraft design.

The results of this study furnish concrete, quantitative guidance for the design of HALE aircraft. For platforms operating at the flight conditions considered in this work (*U = 25* m/s, $h = 20{,}000$ m), the parametric sweep allows the qualitative regimes discussed above to be expressed as explicit criteria, summarised in Table 6. Because the flexibility parameter *σ * maps monotonically onto the trimmed tip-deflection ratio, the criteria are stated both in terms of *σ * and in terms of the directly observable tip deflection normalised by the semi-span, $\delta _{\textrm{tip}}/L$; the latter form is transferable across configurations to leading order and is the recommended screening metric in early design. The boundaries are those of the present model and flight condition; their transfer to other configurations should be through the deflection ratio and the divergence-proximity parameter $q_\infty /q_D$ rather than through *σ * itself.

In practical terms, the workflow implied by Table 6 is as follows. During conceptual design, a linear static aeroelastic estimate of $\delta _{\textrm{tip}}/L$ at the cruise dynamic pressure suffices to place a candidate configuration in one of the regimes. Configurations falling in the first regime can be cleared with conventional linear tools and decoupled autopilot design. In the second regime, structural sizing loads and trim drag must be computed from a nonlinear static analysis, but linearised stability tools remain acceptable provided the linearisation is performed about the deformed trim state—the undeformed-state flutter analysis is conservative (Trim and flutter speed variation with flexibility section) and may be retained deliberately as a conservative screen. The third regime lies beyond the validity of the present model: the trimmed wing is stalled and, further on, super-divergent, so the strip-theory results there are not physical predictions. In design terms this regime is a signal to stiffen the configuration back into the second regime; should such flexibility genuinely be required, its analysis demands a higher-fidelity, stall-capable aerodynamic model together with a proper flight-dynamics and control formulation, since a coupled model with a representative control law would then be indispensable and the load maximum over a gust event would be governed by the flight-dynamic response rather than by the direct bending alleviation alone (Gust response: rigid vs. flexible section).

## Limitations

The scope of validity of the present results is bounded by the modelling assumptions adopted, and these are collected here explicitly.

*Aerodynamic model.* The two-dimensional strip theory utilised in this study delivers acceptable accuracy for the moderate angles of attack that characterise HALE cruise and gust conditions, but it neglects three-dimensional induced-flow effects beyond the parabolic drag-polar correction, tip effects, and spanwise flow. For off-design scenarios involving high angles of attack ($\alpha > 10^\circ$), flow separation, or dynamic stall, three-dimensional or viscous aerodynamic methods such as UVLM [11], CFD [19], or semi-empirical dynamic-stall models of the kind employed by Arena et al. [10] become necessary. Strip theory tends to overpredict unsteady aerodynamic loads, which produces conservative (early) flutter predictions relative to UVLM (Table 5) but may be non-conservative for high-incidence loads where stall intervenes. A systematic evaluation of the influence of aerodynamic modelling fidelity on manoeuvring flexible aircraft predictions is given in [17]. The gust model is likewise idealised: the discrete gust is applied uniformly across the span, so spanwise gust-penetration and coherence effects are excluded from the parametric conclusions.

*Structural model.* The wing is represented as a one-dimensional geometrically-exact beam with an isotropic, uncoupled cross-section ($\boldsymbol{S}_{12}=\boldsymbol{0}$); chordwise (camber) deformation, cross-sectional warping beyond the standard torsion constant, and bend–twist elastic coupling characteristic of composite construction are not represented. Structural damping is deliberately neglected (Structural model: geometrically-exact beam theory section); the computed flutter speeds and post-gust decay rates are therefore conservative, and configurations with significant inherent damping would exhibit correspondingly larger margins.

*Parametric idealisation and range of validity.* The flexibility sweep scales the entire stiffness matrix by a single factor *1/σ * while holding the mass distribution fixed. This deliberately isolates the stiffness effect, but it does not correspond to a realisable structural redesign, in which stiffness and mass would co-vary; the *σ *-thresholds of Table 6 should therefore be read through the tip-deflection ratio $\delta _{\textrm{tip}}/L$, which remains meaningful across configurations, rather than as absolute stiffness prescriptions. The trim-based results (deformation, trim, pre-stressed flutter, and gust loads) are reported only over the range in which the trimmed equilibrium is physically valid, $\sigma \lesssim 1.25$ (tip deflection up to 72% of the semi-span): beyond this the required trim incidence exceeds aerofoil stall, and for $\sigma \gtrsim 2.2$ the flight speed exceeds the linear torsional divergence speed, so the attached-flow strip model—which represents neither stall nor divergence—is outside its validity, and the deeply folded equilibria it returns there are not physical. The undeformed flutter analysis, by contrast, is a linear calculation independent of any trimmed state and remains valid across the full stiffness range. The trim idealisation (Nonlinear trim procedure section) closes the pitching-moment balance with a constant control couple rather than a modelled elevator deflection; a specific elevator implementation would modify the short-period characteristics quantitatively, though not the flexibility trends that are the subject of this study.

*Longitudinal flight-dynamic representation.* This is the most important limitation to state plainly. The framework was constructed and validated for the aeroelastic response, and its free-flight longitudinal model is deliberately simplified: the aircraft pitch attitude is carried by a root feathering (torsion) mode rather than an independent rigid-body rotation, and the thrust and trimming couple are held constant with no thrust–speed or pilot feedback. A direct consequence is that the model does not reproduce the textbook rigid-aircraft longitudinal modes: linearising the nearly rigid configuration ($\sigma \rightarrow 0$) yields no lightly damped oscillatory phugoid near the expected $\sqrt{2}\,g/U \approx 0.55$ rad/s, but instead slow aperiodic roots, because at low *σ * the feathering “pitch” degree of freedom is dominated by the (stiffened) wing torsional stiffness rather than by the aerodynamics. The longitudinal flight-dynamic eigenvalues of this model are therefore not quantitatively reliable, and for that reason the paper reports the flexibility–phugoid trend only qualitatively (Flexibility effect on flight dynamic response section), consistent with the dedicated study of Patil and Hodges [5], and defers eigenvalue-level flight-dynamic predictions to a model with a proper rigid-body attitude degree of freedom and a representative propulsion/control law. The aeroelastic results—natural frequencies, flutter and divergence speeds and their variation with flexibility, static deformation, and the direct gust-passage loads—are computed by the clamped-wing and nonlinear-static modules and are independent of this limitation.

*Other flight-mechanics scope.* The parametric results address the longitudinal dynamics of a symmetric configuration; lateral-directional stability at large static dihedral is discussed only qualitatively (Effect of lift vector rotation on flight dynamics section), and asymmetric gusts, control-surface failures, and manoeuvring flight are outside the present scope.

## Conclusions

A coupled aeroelastic–flight dynamic framework has been developed and employed in a systematic parametric investigation of how structural flexibility affects the flight dynamics of high-aspect-ratio wings. The framework integrates a geometrically nonlinear beam model, unsteady strip-theory aerodynamics with augmented-state lag terms, and rigid-body flight dynamics within a monolithic coupling scheme that treats all inter-disciplinary interactions implicitly. The principal conclusions are as follows. Structural flexibility fundamentally transforms the trim conditions, stability characteristics, and dynamic gust response of high-aspect-ratio wings; the trim angle of attack varies non-monotonically with flexibility—torsional wash-in initially reduces it, while increasing wing bending redirects lift through the lift-vector rotation effect and drives it upward, the required thrust rising correspondingly with the induced drag. These trim results hold over the range in which the trimmed equilibrium is physically valid ($\sigma \lesssim 1.25$, tip deflection up to 72% of the semi-span); beyond it the required incidence exceeds aerofoil stall and, further on, the flight speed exceeds the torsional divergence speed, so the model is applied outside its validity and those states are not interpreted as flyable. Consistent with the results of Patil and Hodges [5], structural flexibility is found to erode longitudinal flight-dynamic (phugoid) stability, confirming that active flight control is essential for very flexible aircraft; the physical mechanism involves both a shift of the effective aerodynamic centre and a reduction in the effective vertical-force lift-curve slope attributable to the bending-induced dihedral. This trend is reported here qualitatively: the present framework is constructed and validated for the aeroelastic response, and its simplified longitudinal free-flight representation does not reproduce the rigid-aircraft phugoid quantitatively, so quantitative flight-dynamic eigenvalues are deferred to a dedicated flight-mechanics model. Flutter speed diminishes with increasing flexibility, but the rate of degradation hinges critically on whether the analysis accounts for the pre-stressed equilibrium, as the geometric stiffening effect of the pre-stress raises the flutter speed above the prediction based on the undeformed analysis by up to about three per cent over the physically valid flexibility range; an aeroelastic mode-tracking analysis shows the flutter mechanism remaining a bending–torsion coalescence that shifts to lower speed and lower frequency as flexibility increases. The coupled framework reproduces the published dynamic benchmarks with good accuracy: natural frequencies within 0.1% of exact beam theory solutions, flutter speed and frequency within 1.5% of established predictions [2, 11], and the torsional divergence speed of Patil and Hodges recovered exactly; the static aeroelastic deflections are conservative with respect to the CFD and UVLM references, a documented consequence of the two-dimensional aerodynamics operating close to the torsional divergence condition. Geometric stiffening imposes a natural limit on deformation during gust encounters, reducing peak tip deflections relative to linear predictions. Flexibility alleviates the direct gust bending loads across the whole span—the peak bending-moment amplification during the gust falls from about 2.8 for the rigid wing to 1.6–1.9 at the nominal flexibility, and the flexible wing’s absolute peak gust moment is lower than the rigid wing’s despite its higher trim moment; moreover, the load maximum over a complete gust event is governed by the subsequent longitudinal motion rather than by the direct response, and its quantification requires a dedicated flight-dynamics model. The stiffness parameter study provides design guidance: linear analysis is acceptable at low flexibility levels, while a fully nonlinear, geometrically consistent formulation is required at higher flexibility levels, and at the highest flexibility levels active flight control is mandatory to stabilise the flexible aircraft. Strip theory is adequate for preliminary aeroelastic design at moderate angles of attack, but three-dimensional aerodynamic methods are recommended for detailed analysis, particularly at high angles of attack and for flutter speed prediction. Finally, fully coupled aeroelastic–flight dynamic analysis is essential for configurations with *σ > 1*, where the interaction among structural deformation, aerodynamic loads, and rigid-body dynamics produces qualitatively different behaviour from that predicted by decoupled approaches, and the effective dihedral created by large wing deformations introduces lateral-directional coupling that cannot be neglected.

Future work will extend the framework to incorporate active control synthesis for phugoid stabilisation [16, 29], gradient-based stiffness optimisation, and coupling with higher-fidelity UVLM and RANS aerodynamics for transonic flexible aircraft applications. The development of reduced-order models [3, 22] suitable for real-time control implementation will also be pursued, along with extensions to asymmetric configurations such as those involving control surface deflections or partial span damage. Experimental validation using wind-tunnel models with calibrated flexibility levels, in the spirit of the Pazy wing benchmark employed by Riso and Cesnik [25], will provide additional confidence in the fidelity of the computational predictions.

## Data Availability

The datasets generated during the current study are available from the corresponding author on reasonable request.

## Code availability

The in-house Python framework used to generate all results, together with the scripts producing every figure and table, is available from the corresponding author on reasonable request.
